# The Evolution of Wisteria Vein Mosaic Virus: A Case Study Approach to Track the Emergence of New Potyvirus Threats

**DOI:** 10.3390/pathogens13111001

**Published:** 2024-11-15

**Authors:** Massimiliano Morelli, Giusy D’Attoma, Pasquale Saldarelli, Angelantonio Minafra

**Affiliations:** Consiglio Nazionale delle Ricerche, Istituto per la Protezione Sostenibile delle Piante, Sede Secondaria di Bari, 70126 Bari, Italy; giusy.dattoma@ipsp.cnr.it (G.D.); pasquale.saldarelli@ipsp.cnr.it (P.S.); angelantonio.minafra@ipsp.cnr.it (A.M.)

**Keywords:** WVMV, wisteria, virus evolution, population genetics, selection pressure, RTDT molecular clock, network inference, high-throughput sequencing, emerging viruses

## Abstract

Wisteria vein mosaic virus (WVMV, *Potyvirus wisteriae*), a virus belonging to the genus *Potyvirus*, is responsible for Wisteria vein mosaic disease (WMD), a severe disease that affects *Wisteria*, a genus of garden plants acclaimed worldwide. Although probably originating in the Far East, WVMV infection was first reported in the US, and subsequently in numerous countries. Following the first molecular detection of an Italian isolate, WVMV Bari, its full-length genome was achieved using NGS barcoding technology. A PhyML phylogenetic analysis, supported by clustering algorithm validation, identified a clear separation between two phylogroups. One major clade comprised WVMV strains isolated from *Wisteria* spp. A second clade grouped three highly divergent strains, at the borderline species threshold, all found in non-wisteria hosts. Relying on a Relative Time Dated Tips (RTDT) molecular clock, the first emergence of WVMV clades has been traced back to around the 17th century. A network inference analysis confirmed the sharp separation between the two host-related phylogroups, also highlighting the presence of potential intermediate variants. Inter-population genetic parameters revealed a very high genetic differentiation in both populations, which was made reliable by statistically significant permutation tests. The migrant number (*N*m) and fixation index (*F*_ST_) evidenced a restricted gene flow and strong population structures. According to the d*N*/d*S* ratio and negative neutrality tests, it was derived that purifying selection at the expense of non-silent variants is underway within WVMV populations. Targeting WVMV evolutionary traits, the present effort raised interesting questions about the underestimated potential of this culpably neglected species to spread in economically relevant crops. The main intention of our study is, therefore, to propose an evolution-based analysis approach that serves as a case study to investigate how other potyviruses or newly emerging viruses may spread.

## 1. Introduction

Wisteria vein mosaic virus (WVMV, *Potyvirus wisteriae*) is a member of the genus *Potyvirus*, family *Potyviridae*. WVMV shares a typical genomic structure with other potyviruses, characterised by a positive-sense single-stranded RNA molecule approximately 10 kb in size [1,2]. Based on pioneering studies in the early 1970s [3,4] and repeatedly confirmed by molecular evidence in recent times [5], WVMV is the recognised causative agent of Wisteria mosaic disease (WMD) [6]. Known to occur in numerous countries worldwide, WMD is currently considered the most serious disease affecting several species of wisteria [7], a genus of woody leguminous plants widely cultivated for ornamental purposes in almost all continents [8].

According to current knowledge, WVMV and the associated disease seem to have originated in the Far East [7,9], which is also the place of origin of the most affected wisteria species (*Wisteria sinensis* and *W. floribunda*) [10]. Nonetheless, numerous reports, especially in the last few years, have attested to its increasingly widespread presence also in western countries, and in particular in Europe [6,7,11,12]. Our recent study has served to substantiate the presence of the virus in Italy with molecular evidence [7], which, relying only on serological methods, had been hypothesised around fifty years earlier [4]. Once the presence of the virus in southern Italy had been proven, other reports followed in the northern regions of the country [12].

Renewed interest in the emergence of the virus over an ever-widening geographical area, combined with concern for the damage WMD can inflict on plant species of great appeal to ornamental nurseries, has alerted several research groups. New molecular data have been made available in GenBank, and only in the last two years, the number of complete WVMV genomic sequences available has almost tripled. The present study contributes to this collective effort. Furthermore, the complete genome of the WVMV isolate Bari, which could not be provided in the previous report, is now available and will be discussed in detail.

Recently, the possible occurrence of WVMV and phylogenetically “allied” viruses of still uncertain taxonomic attribution in other herbaceous species of the Fabaceae family [5,13] has raised further points of discussion on both their potential to spread and the evolutionary modes of their emergence. The ability of WVMV to be dispersed without spatial constraints by the circulation of untested propagation material [7,9], combined with its proven transmissibility through different aphid species [14], increases the risks of inter-specific spread and makes the effort to characterise its past and future evolutionary dynamics even more interesting.

In our previous report [7], preliminary evidence was gathered from the data available at the time on the usefulness of evolutionary analysis (based on genetic diversity and population dynamics) to understand the dynamics of WVMV emergence. There is now broad consensus that the emergence and spread of new viral diseases are strongly influenced by viral trafficking in an inter-host ecological context [15,16]. In turn, this readjustment to a broader ecological context that changes over time makes evolutionary mechanisms a signature evident in viral sequences [17], especially when transmitted by vectors, as is the case with potyviruses [18] and WVMV in particular.

The present work, building on the achievement of the complete genome sequence of the WVMV isolate Bari found in Italy, extends the effort begun in the foregoing study. This study aims to attempt a phylogenetic and evolutionary reconstruction of the relationships between the currently known isolates of the virus in order to trace the pathways of its emergence. Although little investigated until recently, from a taxonomic point of view and in terms of its ecological implications, WVMV constitutes a very peculiar subject within the potyviruses. Following the detection of WVMV in Italy, the European and Mediterranean Plant Protection Organization (EPPO) recently included this virus in its periodical alert report, pointing out its potential threat to the nursery market [19].

We therefore consider WVMV to be the ideal candidate to propose here an evolution-based approach, which also makes use of population genetics tools, with the intention of providing a case study to trace the emergence of new viral threats in the genus *Potyvirus* and beyond.

## 2. Material and Methods

### 2.1. Virus Plant Source

**Figure 1:**
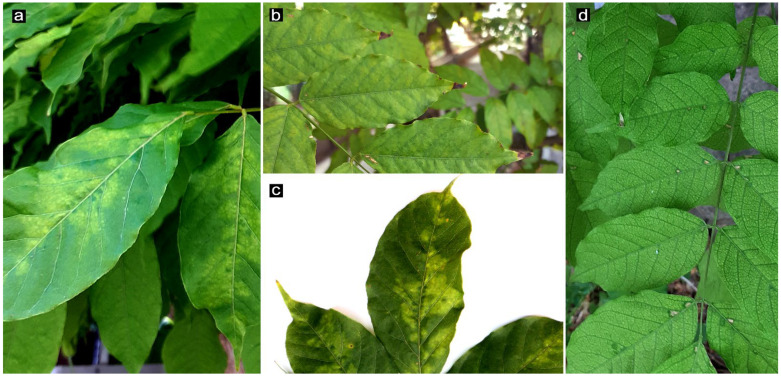
Wisteria mosaic disease (WMD) symptoms observed in early summer 2022 on the leaves of a Chinese wisteria (*Wisteria sinensis* Sims) ‘Sweet’ tree infected with Wisteria vein mosaic virus (WVMV, *Potyvirus wisteriae*) isolate Bari. The compound leaves showed mosaic spots and irregular light green and yellow mottling near the veins (**a**,**b**). In some cases, the symptoms evolved into more extensive chlorosis, ring spots, necrotic patches and leaflet distortions (**c**). The leaflet of a symptomless, healthy tree is shown in (**d**) for reference.

As reported in our previous study [7], a severely WMD-affected Chinese wisteria tree (*W. sinensis* Sims), variety ‘Sweet’, had been identified in a garden in the urban area of Bari (Apulia, southern Italy). The tree had been found infected with an isolate of WVMV, which was named Bari, from the site of discovery. In 2022, after winter dormancy and flowering in spring, a reappearance of symptoms was observed in early summer during foliage growth. Irregular, light green to yellow patterns or mottling appeared on the leaves. Mosaic spots of irregular shape and size appeared adjacent to the leaf veins, sometimes progressing into more extensive chlorosis. Slight distortions, wrinkling or lateral twisting were also occasionally observed on the leaflets (Figure 1).

To further characterise and achieve the complete genome sequence of the WVMV isolate Bari, already identified in association with the symptoms observed in the previous vegetative season, symptomatic leaves were sampled randomly from different portions of the tree canopy.

### 2.2. Double-Stranded RNA Purification, Complementary DNA Synthesis, Random Amplification and High-Throughput Sequencing

Purification of double-stranded RNAs (dsRNAs), synthesis of complementary DNA (cDNA) and random amplification prior to high-throughput sequencing (HTS) were performed following the procedure described by Marais et al. [20], with slight modifications. Briefly, dsRNAs were purified from 0.5 g of pooled leaf blade samples by double-step batch chromatography with cellulose fibrous powder (CF11, Whatman plc, Little Chalfont, UK). Following digestion with DNase I and RNase A (Thermo Fisher Scientific, Waltham, MA, USA), after ethanol precipitation, dsRNAs were visualized in 1.2% Tris/Borate/EDTA (TBE) agarose gel electrophoresis. After heat denaturation, dsRNAs were reverse transcribed in the presence of 2 mM PcDNA primer (5′-TGTGTTGGGTGTGTTTGGN_12_-3′) [21], 0.5 mM dNTPs, 10 mM DTT, 1 U of RNaseOUT™ RNase Inhibitor, 200 U of SuperScript™ II Reverse Transcriptase and 1X first-strand buffer, prior to final digestion with RNase H. All reagents were from Thermo Fisher Scientific (Waltham, MA, USA).

The obtained cDNA was used as a template to perform a PCR-based random amplification in the presence of 1 µM multiplex identifier (MID) primer (5′-**CAAGTCAA**TGTGTTGGGTGTGTTTGG-3′, MID tag in bold), 0.25 mM DNTPs, 0.3 U of DyNAzyme™ II DNA polymerase and 1X reaction buffer (Thermo Fisher Scientific, Waltham, MA, USA). Cycling parameters were those reported in the original protocol [20]. After primer removal with the QIAquick PCR Purification Kit (Qiagen N. V., Venlo, The Netherlands), the use of MID adaptor tagging allowed amplicon pooling with other samples, unrelated to this study. Indexed libraries were prepared at the Genomix4Life Srl facility (Salerno, Italy) using the TruSeq™ Stranded mRNA kit (Illumina^®^ Inc., San Diego, CA, USA) and sequenced in multiplexed format (2 × 150 bp paired-end) on a Nextseq^®^ 500 system (Illumina^®^ Inc., San Diego, CA, USA).

### 2.3. High-Throughput Sequencing Data Analysis

The HTS data were uploaded to the Galaxy web platform [22] to perform all subsequent analyses. The raw Illumina^®^-sequenced reads were trimmed and filtered with fastp version 0.23.2 [23]. Key quality metrics before and after processing were obtained with MultiQC version 1.11 [24]. The quality-trimmed reads were mapped with Bowtie2 version 2.5.0 [25] against the *W. sinensis* RefSeq chloroplast genome (NC_029406.1). SAMtools view version 1.15.1 [26] was used to filter the data and only those reads that were not mapped to the available host reference were retained.

The de novo assembly was built with the MEGAHIT version 1.2.9 ultra-fast single-node assembler [27] using default settings. To allow local BLAST searches of the assembled contigs, two databases consisting of viral RefSeq plus neighbour nucleotides and RefSeq proteins, respectively, retrieved from GenBank, were created using the NCBI BLAST+ version 2.14.1 [28] makeblastdb option. The origin of the query contigs was checked against the nucleotide and protein databases with NCBI BLAST+ blastn and blastx search tools. The expected value (E-value) cutoff was set at 0.001. Contigs showing alignment hits shorter than 100 nt were excluded from the analysis. The BLAST results were manually edited with Microsoft Office Excel^®^ 2016 (Microsoft Corporation, Redmond, WA, USA) and the R package tidyverse version 1.3.0 [29], to include the taxonomic information reported in the ICTV Virus Metadata Resource (VMR) database [30].

Based on the contigs retrieved, the consensus genome sequence of the WVMV isolate Bari was refined with ivar consensus version 1.4.2 [31], with the minimum frequency threshold set at 0.7, minimum quality score threshold to count bases at 20 and minimum depth to call consensus at 2.

### 2.4. Genome Sequence Assembly and Annotation

Validation of the 5′- and 3′-terminal sequences of the WVMV Bari genome identified by bioinformatic analysis was carried out with 5′- and 3′-rapid amplification of cDNA ends (RACE) experiments using the 5′/3′ RACE Kit, 2nd Generation (Roche^®^ Life Sciences, Basel, Switzerland), according to the manufacturer’s protocol. The sequence-specific primers wv5r 5′-TACAGGACGTGACCCAACA-3′ (this study) and wvcpF 5′-TGTTGTGARTCAGTTTCTCTRC-3′ [7] were used to amplify the 5′ and 3′ ends, respectively. RACE amplicons, cloned into the pSC-A-amp/kan vector (StrataClone PCR cloning kit, Agilent Technologies Inc., Santa Clara, CA, USA), were custom sequenced (Macrogen Europe, Milan, Italy) and the accuracy of HTS sequencing was confirmed with 100% identity.

Genome annotation, prediction of open reading frames and translation into amino acid sequences were carried out with the CLC Genomics Workbench 3.6.5 (CLC bio, Aarhus, Denmark). The analysis of structural and functional domains conserved within the protein sequences was conducted with the Conserved Domain Search service (CD-Search) tool [32]. The cleavage sites within the polyprotein potentially encoded by WVMV Bari were predicted based on the conserved motifs described for potyviruses [33] and using the ExPASy PeptideCutter tool [34].

### 2.5. Read Coverage and Variant Call Analyses

The raw HTS reads were mapped back to the derived genome sequence of WVMV Bari with the above-mentioned Bowtie2 tool. Read coverage over the entire genome was computed with QualiMap BamQC version 2.2.2c [35]. Mapped reads and coverage graphs were visualized using Geneious Prime^®^ version 2022.2.2 (Biomatters Inc., Auckland, New Zealand) and Jbrowse genome browser version 1.16.11 [36]. Single nucleotide variant (SNV) call analysis was carried out using ivar version 1.4.2 [31]. To avoid inaccurate calling, only variants showing a significant *p*-value (<0.05) were retained.

### 2.6. Sequence Comparison and Phylogenetic Analysis

A dataset of the complete coding region of 147 available potyvirus sequences classified as RefSeq was retrieved from the NCBI virus database [37]. The corresponding sequences of six assigned or putative species not classified as RefSeq but available in GenBank, viz peanut stripe virus blotch (PStV, U05771), Calla lily latent virus (CLLV, EF105298), Passiflora chlorosis virus (PaCV, OL584353), Uraria mosaic virus (UMV, LC477217), Passiflora foetida virus Y (PfVY, LC466655) and kudzu chlorotic ring blotch virus (KudCRBV, OQ148665) were also included in the list, along with the WVMV isolate obtained in this study, and one rymovirus isolate, ryegrass mosaic virus (RGMV, NC_001814), added as an outgroup. Nine other WVMV isolates, for which the complete coding region sequence was available in the NCBI virus database, were also included in our dataset and used for intraspecific comparisons. Furthermore, three additional isolates of WVMV, for which at least the complete coat protein (CP) gene was available, were considered for further analyses, together with eight isolates with only a partial fragment available and straddling the nuclear inclusion body b protein (NIb) and CP genes (Table S1).

Nucleotide or translated sequences were subjected to multiple pairwise alignment using fast Fourier transform (MAFFT) [38]. Two-dimensional diversity matrices were generated using the Sequence Demarcation Tool (SDT) software, version 1.2 [39]. Phylogenetic analyses were carried out using the PhyML with Smart Model Selection (SMS) tool, available at the Next Generation Phylogeny.fr web service [40]. PhyML is a phylogenetic inference method based on the maximum Likelihood (ML) approach [41]. MAFFT alignments were refined with Block Mapping and Gathering with Entropy (BMGE) version 1.2 [42], allowing for a phylogenetic reconstruction inferred by PhyML v. 3.3. Starting tree topologies were computed using the BioNJ algorithm [43] and rearranged with a parsimony-based search relying on Subtree Pruning and Regrafting (SPR) topological moves [41,44]. The most appropriate substitution models were selected using the fully integrated SMS tool [45] and based on the Akaike Information Criterion (AIC) [46]. Branch support was measured according to the Shimodaira–Hasegawa (SH)-like method [41] or based on bootstrap nonparametric analyses [47] with 1000 replicates.

The inferred trees were rendered using the Newick display utility [48] and FigTree version 1.4.4 graphical viewer [49] prior to final editing with the Interactive Tree of Life (iTOL) software, version 6.5.8 [50].

Statistically significant clusters were identified in each tree using the TreeClus algorithm, which uses a dissimilarity measure calculated from the distribution of branch lengths to divide and cluster phylogenetic topologies [51]. The dissimilarity threshold was set to a minimum value of 50%.

### 2.7. Recombination Analysis

The MAFFT-aligned nucleotide sequences of the complete coding region were screened for the presence of recombination events using the Recombination Detection Program, version 4.101 (RDP4) [52]. Together with the eleven available WVMV isolates, KudCRBV isolate Ack01 was also included in the analysis. A full exploratory recombination scan was performed using all nine detection methods implemented in RDP4 (RDP, GENECONV, Chimaera, MaxChi, BootScan, SiScan, PhylPro, LARD, 3Seq). A Bonferroni-corrected *p*-value cut-off of 0.05 was considered significant, and evidence of recombination was accepted if supported in at least three different methods of detection.

### 2.8. Dating Analysis

Since the collection dates of the eleven fully sequenced WVMV isolates available in the NCBI database were known, they were used to estimate a time tree. Divergence times were inferred relying on the Relative Time with Dated Tips (RTDT) method [53], which uses a relaxed molecular clock built on the algebraic relative rate framework [54]. The time-scaled tree was computed in the MEGA 11 program [55] by applying the RTDT analysis to an ML phylogenetic tree based on the complete coding region of the eleven WVMV isolates available. The KudCRBV isolate Ack01 was also included, together with the watermelon mosaic virus (WMV, *Potyvirus citrulli*) isolate Fr (NC_006262), used as an outgroup. The most suitable substitution model was selected by the SMS tool, according to the AIC criterion.

### 2.9. Network Inference Analysis

To further investigate the evolutionary relationships between the known isolates of WVMV, an Integer Neighbour-Joining (NJ) network was created using the PopART (Population Analysis with Reticulate Trees) software version 1.7 [56]. The KudCRBV isolate Ack01 was also included in the analysis. Network inference methods [57] are now widely used to reconstruct the evolutionary links within virus populations, as they allow relationships among isolates to be displayed as a descriptive reticulation, with a resolution at the scale of a single nucleotide [58]. The MAFFT-aligned nucleotide sequences of the complete coding region were used in the analysis, by selecting the ‘‘Integer Neighbour-Joining Net’’ option, with default settings.

### 2.10. Analysis of Genetic Differentiation and Population Dynamics

The dataset comprising the eleven WVMV isolates and KudCRBV Ack01 isolate used in previous analyses was also exploited to investigate the extent of genetic differentiation and gene flow between the different virus populations. The viral sequences were grouped according to their host of provenance, thus identifying a major group of 9 sequences from *Wisteria* spp. and a minor group of 3 sequences isolated from non-wisteria hosts.

Genetic differentiation indices and population genetic parameters were computed with the DNA Sequence Polymorphism (DnaSP) software version 6.12.03 [59], based on MAFFT multiple alignment of the complete nucleotide sequences of the coding region.

The gene flow and differentiation between the two populations were assessed by pairwise comparisons and estimated in terms of fixation index (*F*_ST_), which evaluates the amount of genetic variance within a subpopulation relative to the overall genetic diversity and relies on Wright’s *F*-statistic [60]. The number of migrants per generation (*N*_m_) was also evaluated and calculated as [1/*F*_ST_) − 1]/4, according to the formula proposed by Slatkin [61].

Inter-population genetic differentiation was also estimated on the basis of the following parameters: the average number of nucleotide substitutions per site between populations (*D*_xy_), the average proportion of nucleotide differences between populations (*K*_xy_) and the number of net nucleotide substitutions per site (*D*_a_). Three permutation-based statistical parameters, i.e., *K*_s_*, Z* and *S*_nn_, were used to further validate the results obtained [62,63], with *α* significance level set at 0.05. Tajima’s *D* [64] and Fu and Li’s *D* and *F* [65] neutrality tests (*α* level: 0.1) were used to challenge the hypothesis of selection forces acting on the polyprotein gene or evolutionary events shaping the population sizes. To analyse selective pressure at the molecular level, the average number of synonymous (d*S*) and nonsynonymous (d*N*) substitutions per site and the d*N* to d*S* ratio (*ω*) were estimated by the Jukes and Cantor (JC) correction, computed according to the simplification indicated by Nei and Miller [66].

Since the CP of potyviruses can be a preferred target for selection [63], the selective pressures acting on the CP cistron were also investigated. The codon-based maximum Likelihood algorithm SLAC (Single-Likelihood Ancestor Counting), implemented on the Datamonkey server [67], was used to evaluate *ω* at each codon site, with default settings. Differently from previous population analyses targeting the full coding region, the SLAC analysis was conducted on a larger dataset (n = 15), which also included WVMV isolates YZ (MK119780.1), Australia (AF484549.1) and Th-W2259, for which only the CP region was available. MAFFT-aligned nucleotide sequences were used as input for the analysis.

## 3. Results

### 3.1. High-Throughput Sequencing Data Analysis

The high-throughput Illumina^®^ sequencing of dsRNA libraries isolated from the *W. sinensis* tree found to be infected with WVMV in our previous study [7] yielded a dataset consisting of 24,274,274 redundant raw reads (BioProject PRJNA1177767). After fastp adaptor trimming and quality filtering, a total of 23,255,300 paired-end reads of 145 bp length (95.8% of the total) were obtained. To enrich the RNA fraction of viral origin, 5,499,394 reads (23.65% of the total) mapped with Bowtie2 to the available host sequence (the complete plastidial reference genome, NC_029406.1) were filtered and removed from the dataset.

After performing de novo assembly of the remaining viral reads using MEGAHIT, a total of 13,353 contigs were generated, ranging in size from 200 to 9941 nt. BLAST iterative analyses against nucleotide and protein viral databases identified 138 contigs showing significant homology to known viral sequences (Table S2). The near totality of these contigs (133 out of 138) showed significant aa identity (42.6 to 100.0%) with members of the genus *Potyvirus* and could be identified as segments of the WVMV Bari genome, as expected.

The BLAST analysis also revealed the potential presence of other viruses by identifying two contigs (410 and 333 nt) homologous to sequences of wisteria badnavirus 1 (WBV1, *Badnavirus wisteriae*), two contigs (130 and 200 nt) to sequences of the genus *Alphaendornavirus* and one contig (397 nt) to sequences of the genus *Deltapartitivirus*. However, the occurrence of these possible co-infections was not investigated further, as it was beyond the scope of this study.

A long contig mapping with 100% query coverage on the genome sequence of the WVMV isolate Ir (MN514947) was identified as the full-length sequence of the WVMV isolate Bari and subjected to further validation. A BLASTN 100% identity consensus was found with regions of the WVMV Bari genome already sequenced by the Sanger method in our previous analyses (OP748400.3, OP381183.1), thus confirming the correct identification of the assembled contig. Bowtie2 remapping, followed by an ivar consensus call, allowed a final consensus sequence to be further refined. The accuracy of the assembly was also validated with RACE analysis of the 5′ and 3′ ends.

### 3.2. Genome Organization of WVMV-Bari

**Figure 2:**
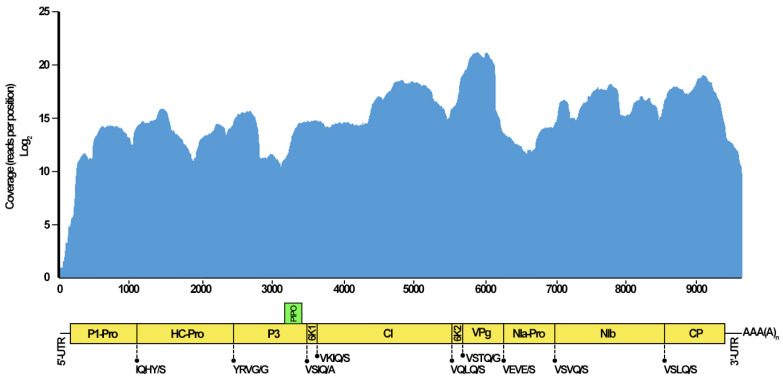
Genomic structure of Wisteria vein mosaic virus (WVMV) isolate Bari, with an indication of the putatively encoded proteins and the sequences of the polyprotein cleavage sites, predicted according to Goh and Hahn [33] and using the ExPASy PeptideCutter tool [34]. The top panel shows the coverage depth (Log_2_ reads per position) obtained by mapping the Illumina^®^ raw reads against the assembled WVMV genome. Read mapping and coverage computation were performed with Bowtie2 version 2.5.0 [25] and BEDTools genome coverage version 2.30.0 [86], respectively. **P1-Pro:** P1 proteinase; **HC-Pro:** helper component proteinase; **P3:** P3 protein; **6K1:** 6K1 protein; **CI:** cylindrical inclusion; **6K2:** 6K2 protein; **VPg:** viral protein genome-linked; **NIa-Pro:** nuclear inclusion proteinase a; **NIb:** nuclear inclusion body b protein; **CP:** coat protein; **PIPO:** Pretty Interesting Potyviridae ORF protein, **5′-3′ UTR:** 5′-3′ untranslated regions.

The complete WVMV Bari genomic RNA was found to be 9694 nt in size, excluding the 3′-poly(A) tail. The 5′ and 3′-UTRs were relatively short, being 163 and 252 nt in length, respectively. The sequence has been deposited in the GenBank database under the accession number OR567543. The genome sequence started with a decanucleotide 5-AAAAUUAAAA similar to the initial nucleotide motifs found in several potyviruses [5]. The highly conserved potybox ‘a’ was identified in the 5′-UTR as A_28_CAACAa_34_ [68]. The 5′ leader sequence contained several CAA triplet repeats, known to be associated with translation enhancement, as described in tobacco mosaic virus (TMV) [69]. The 5′-UTR had a higher content of AU (60.3%) than GC (30.7%), as reported in other potyviruses [9,70]. The 3′-UTR, whose secondary structure might be involved in genome replication [71], was AU-rich (60.7%), like many other potyviruses [1]. The genome contained a single large ORF (9279 nt) which started with the first in-frame AUG_164–166_, presumed to be the initiation codon of the polyprotein translation, and ended with a UAA_9440–9442_ termination codon. The putatively encoded polyprotein (3092 aa), with an estimated molecular mass of 353.627 kDa, is presumably cleaved by three self-encoded virus proteases to yield ten mature functional proteins. Based on an in silico analysis suggesting the cleavage sites, as shown in Figure 2, the ten mature products could be identified, proceeding from the N- to C-terminus, as: P1 proteinase (P1-Pro), helper component proteinase (HC-Pro), P3 protein (P3), 6K1 protein (6K1), cylindrical inclusion (CI) protein, 6K2 protein (6K2), viral protein genome-linked (VPg), nuclear inclusion proteinase a (NIa-Pro), nuclear inclusion body b protein (NIb) and coat protein (CP) (Table S3). The cleavage sites were mostly in consensus with those identified in the polyprotein of the WVMV reference isolate Beijing (NC_007216.1). However, the cleavage site at the P3/6K1 junction was identified as _1118_VSIQ/A_1122_, unlike Beijing where proteolytic cleavage should occur at a VSMQA site. This substitution is not frequent in the other WVMV isolates but is present in WVMV Ir (MN514947.1). The cleavage site identified at the 6K1/CI junction (_1170_VKIQS_1174_) also differed from the reference found in WVMV Beijing (VKAQS), but interestingly this was a feature shared with other WVMV isolates, including the divergent Ce-JH (LC729727). In both 6K1/CI and CI/6K2 sites, the occurrence of the amino acid isoleucine (I) should be considered a distinguishing feature, as it is reported with a frequency of less than 4% among the consensus sequences of the potyvirus NIa-Pro cleavage sites described by Goh and Hahn [33].

Conserved domains homologous to other known potyviruses were identified by CD-Search, including the catalytic core domain of RNA-dependent RNA polymerase (RdRp) in the family *Potyviridae* (cd23175), a helper component proteinase found in the polyprotein of potyviruses (cl20022, pfam00851), the potyvirus coat protein domain (cl02961, pfam00767), a *Potyviridae* polyprotein domain (cl07169, pfam08440) and a C4 family peptidase present in the nuclear inclusion protein of potyviruses (cl24133, pfam00863) (Table S4). Several highly conserved motifs with known functions predicted in other potyviruses were identified within the putative coding region of WVMV Bari. The motifs H_230_-(8X)-D/E_239_, G_269_-(X)-SG_272_, I/V_293_RGR_296_ were found in P1-Pro protein, which may represent the active catalytic serine sites responsible for protein self-proteolysis [1,72,73]. The conserved motifs C_660_-(72X)-H_733_, known to be potentially responsible for protease activity [1] and C_608_CCVT_612_, which together with the CP motif R_2857_-(45X)-D_2903_ is involved in virus long-distance movement [74], were identified in HC-Pro. Three amino acid motifs, K_370_LSC_373_, F_497_RNK_500_ and P_626_TK_628_, which are reported to be essential in aphid-mediated potyvirus transmission [5,74], were also identified in Hc-Pro. The triad of amino acids his-asp-cys, situated in the context of the conserved motif H_2096_-(34X)-D_2131_-(69X)-C_2201_, was identified in NIa-Pro where it represents the protease active site [75,76]. The nucleotide-binding motif _1258_GAVGSGKST_1266_ [77] was found in CI, which also carried the RNA helicase domains _1278_VLLEPTRPL_1287_, _1347_DECH_1350_, _1374_KVSAT_1378_, _1425_LVYV_1428_, _1476_VATNIIENGVTL_1487_ and _1520_GERIQRLGRVGR_1531_ [78,79]. A tyrosine residue (Y_1924_) was found in the conserved motif _1923_MYGV_1926_ which is required for linking VPg to the 5′-terminus of the potyviral RNA [1,77]. The conserved RdRp motifs _2536_VYCHADGSQFDSSLT_2550_, which is crucial for recognising and binding nucleoside triphosphates and metal ion cofactors [80,81], and _2603_SG-(3X)-NT-(30X)-GDD_2613_, a feature shared among positive-stranded RNA viruses [72], were identified in the NIb protein. The DAG triplet was found at two repeated sites (aa 2820–2822 and 2860–2862) in the N-terminal region of CP. This motif is thought to be critical for the interaction of CP with HC-Pro sites involved in potyvirus transmission by aphids, as mentioned previously [74,82]. In CP, the conserved _2828_NAG_2830_ motif was also identified, which is a feature shared with other aphid-transmitted potyviruses [78,83] although less frequent than the DAG motif [84].

A second overlapping ORF, putatively coding the recently identified *pipo* (Pretty Interesting Potyviridae ORF) protein [85] was detected, embedded within the P3 cistron. This small CDS (nt 2941–3168) starts within the highly conserved GA_6_ motif [77] and putatively codes for a 75 aa product (+3 reading-frame, relative to polyprotein).

### 3.3. Read Coverage and Variant Call Analyses

Bowtie2 *a posteriori* mapping of the raw reads against the assembled WVMV Bari genome revealed that 45% (10,460,864 out of 23,255,300) of the total reads were derived from this genome and that these yielded a 100% complete genome coverage, with a mean of 158,693 reads per position. A significant peak in the read count, with coverage even exceeding 1.5 million reads per position, was identified in a 321 bp-long segment (nt 5809–6129) located in the region coding for the VPg protein. By contrast, a low coverage (<100X) was found in a portion (nt 1–213) encompassing the whole 5′-UTR and the region coding for the N-terminus of the P1 protein. A coverage <100X was also found at the very 3′-teminus (nt 9688–9694) (Figure 2). The unevenly distributed coverage along the genome, being higher in the C-terminal half, was in line with previous reports for other *Potyvirus* species, including Catharanthus mosaic virus (CatMV, *Potyvirus catharantessellati*) [87] and passion fruit woodiness virus (PWV, *Potyvirus passiflorae*) [88].

To annotate intra-isolate variability, a variant call analysis was performed. Based on the output of the ivar tool, 1116 potential single nucleotide variants, which showed a significant *p*-value, were identified within the CDS. The predicted frequency of alternative variants with respect to the consensus sequence, calculated for a given position, was on average 8%, ranging from 3 to 52%. About 50% of these polymorphisms could consist of potential amino acid residue changes (565 purine/pyrimidine transversions and 262 missense transitions, distributed in 305 amino acid sites). In contrast, about 23% of SNVs (254) were identified as synonymous silent mutations. A low percentage, about 3% (35) were identified as nonsense mutations, potentially originating premature stop codons. The highest number of potential SNVs was found in the NIb region (203), while their potential occurrence seemed very rare in the 6K1 coding sequence (3) (Table S5).

### 3.4. Sequence Comparison and Phylogenetic Analysis

**Figure 3:**
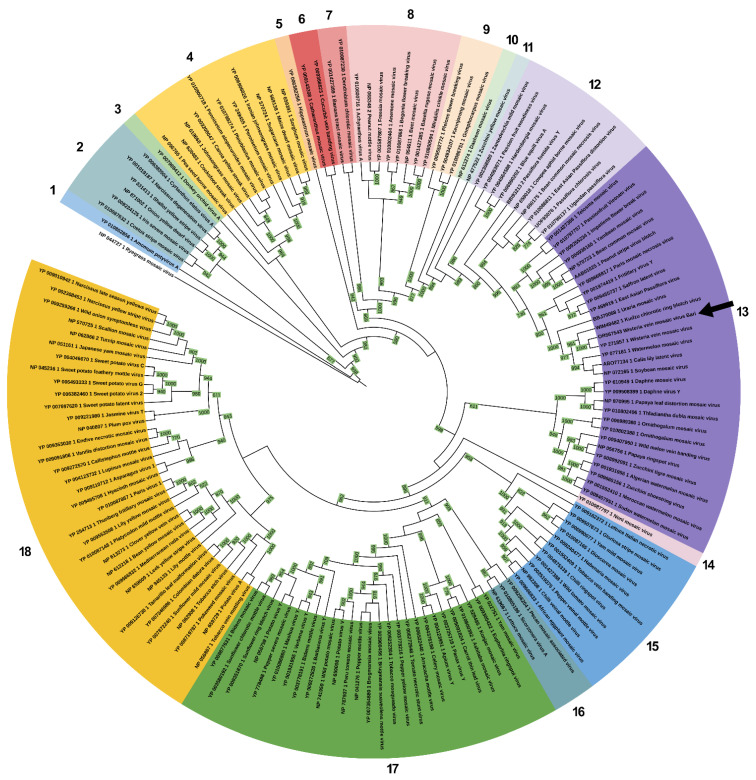
Radial cladogram based on the complete sequence of the polyprotein (2632–3493 aa) of 155 isolates representative of 154 putative or assigned species of the genus *Potyvirus*. Phylogenetic reconstruction, based on the Maximum Likelihood (ML) approach, was performed with the PhyML/SMS workflow available at the NGPhylogeny web service. Tree editing was carried out with Interactive Tree of Life (iTOL) software, version 6.5.8. The branch length is unscaled. Bootstrap branch support was computed over 1000 replicates. Bootstrap values higher than 500 are shown above the branches. The tree was rooted using ryegrass mosaic virus (RGMV, *Rymovirus lolii*, genus *Rymovirus*) as an outgroup. A total of eighteen clades were found, based on a cluster analysis performed with the TreeClus algorithm [51], with a dissimilarity threshold of >50%. Distinct clades are numbered from 1 to 18 and highlighted in different colours. The arrow indicates the WVMV isolate Bari, the object of this study. All sequences are identified by their accession number and species name. Detailed information on individual isolates can be found in Table S1.

A phylogenetic reconstruction of the relationships of WVMV Bari with 154 other known potyvirus species was attempted, based on the complete amino acid sequence of the polyprotein. Phylogenetic analysis was conducted using the PhyML SMS method. An LG substitution matrix [89] with gamma-distributed rates across invariant sites (+*Γ*+I) and +F equilibrium frequencies showed the lowest AIC score (496.822) and was used to infer the tree phylogeny. A TreeClus clustering algorithm analysis identified 18 distinct lineages among the potyvirus species (Figure 3). WVMV Bari was placed, together with the reference isolate Beijing, in a large clade that included 28 other species (Figure 3, clade 13), and was usually reported as the “bean common mosaic virus (BCMV, *Potyvirus phaseovulgaris*) supergroup” in previous studies [90,91]. Interestingly, WVMV Bari and the type isolate of the species occupied a sub-clade well supported by the bootstrap value, together with the recently discovered KudCRBV, which could represent a distinct but closely related species [5]. Since the taxonomic relationship of KudCRBV with WVMV is currently still in dispute, we decided to include the KudCRBV isolate Ack01 (OQ148665) in the subsequent analyses.

MAFFT pairwise alignment of the complete polyprotein CDS revealed that WVMV Bari shared 76.9–98.7% nt and 82.8–99.0% aa sequence identity with the ten WVMV isolates with a complete genome available in the NCBI database (Figure S1). The sequence identity with the RefSeq WVMV Beijing (86.1% nt, 91.9% aa) was well above the species demarcation threshold. However, WVMV Bari showed the highest values of identity with the two European isolates DSMZ_PV-1105 (OQ731912; 98.7% nt, 99.0% aa) and DSMZ_PV-1026 (OQ993365; 98.7% nt, 99.0% aa), as well as with the Iranian isolate Ir (98.6%, 99.0%). WVMV Bari shared the lowest identity values with the two South Korean isolates Ce-JH (76.9% nt, 83.3% aa) and JEBU-p (MT603851; 77.0% nt, 82.6% aa). It is worth mentioning that identity values in the same range as these divergent isolates were found by comparing WVMV Bari with KudCRBV Ack01 (77.0% nt, 82.8% aa).

Sequence comparison was also carried out for the evolutionarily relevant CP gene, and in this case, it was possible to include three other WVMV isolates, for which only this region was available in GenBank. In the CP region, WVMV Bari shared the highest identity with the German isolates DSMZ_PV-1105 or DSMZ_PV-1026 (99.7% nt, 100% aa) and the two Iranian isolates Ir (99.1% nt, 100.0% aa) and Th-W2259 (MH558668) (98.9% nt, 100% aa), all exceeding the identity values measured with the reference isolate Beijing (88.1% nt, 96.8% aa) (Figure S2). Again, WVMV Bari CP shared the lowest identity values with the South Korean isolates JEBU-p (77.4%, 79.9% aa) and Ce-JH (78.2% nt, 81.3% aa), consistent with those shared with KudCRBV Ack01 (78.2% nt, 80.6% aa).

The P1-Pro gene was used for further sequence alignment analyses, as it is often associated with the highest variability within the potyvirus genome [73]. In accordance with expectations, heterogeneous identity values were found in this region. As for WVMV Bari, it showed an nt identity value in the 91.1–97.5% range (88.6–98.1% aa) with the majority (7 out of 12) of isolates included in the analysis. However, this value dropped considerably in comparison with the divergent WVMV isolates Ce-JH (57.7% nt, 46.7% aa) and JEBU-p (57.8% nt, 46.1% aa), with KudCRBV Ack01 (57.2% nt, 46.9% aa) and interestingly also with the RefSeq WVMV Beijing (68.4% nt, 60.7% aa) (Figure S3a). The divergence with WVMV Ce-JH, WVMV JEBU-p and KudCRBV Ack01 was even more pronounced if the comparison was limited to the N-terminal region (aa 1–100) (identity range 39.2–40.7% nt, 29.1–32.0% aa), often reported as a hypervariable region in the potyvirus polyprotein [92]. By comparison, in the same region, WVMV Bari showed nucleotide sequence identity values between 92.0% (88.0% aa) and 97.3% (96.0% aa) with the remaining WVMV isolates, except for Beijing (68.0% nt, 56.0% aa) (Figure S3b).

In the course of writing, the complete sequence of another Italian isolate of WVMV (Gli2, PP835448) was made available in the NCBI virus database. Based on the available information, the virus was isolated in 1996, presumably in Northern Italy, from *W. floribunda*, then freeze-dried and stored until the recent sequencing. Based on the whole CDS comparison, Gli2 shared a 98.7% nt (98.9% aa) identity with WVMV Bari, slightly less than the two very similar German isolates DSMZ_PV-1105 and DSMZ_PV-1026 (Figure S1).

In addition, following our first report of the presence of WVMV Bari in Italy [7], Pedrelli et al. have found eight new WVMV Italian variants infecting some *W. sinensis* plants in Liguria (Central-Northern Italy) [12]. Unfortunately, the authors only made the sequences of a partial fragment between the 3′ end of the NIb gene and the 5′ CP gene available, and it was not possible to include these eight isolates in our CDS analysis. However, their sequence identity values were compared with WVMV Bari based on the available 525 nt-long fragments, and it was found that they were in the range of 96.6–99.8% nt (98.9–100% aa), with one isolate (Sar 5, OM417219.1) showing lower similarity (87.4% nt, 94.3% aa) (Figure S4). As for the Gli2 isolate, its sequence similarity with these isolates did not exceed 98.7% nt (98.3 aa).

A more in-depth phylogenetic analysis of the relationships of WVMV Bari with interspecific isolates and with KudCRBV Ack01 was attempted. A new phylogenetic tree was designed based on the aa sequence of the polyprotein and the best-fitting Jones–Taylor–Thornton (JTT) model [93] with +Γ+I decoration (AIC score 33881.853).

**Figure 4:**
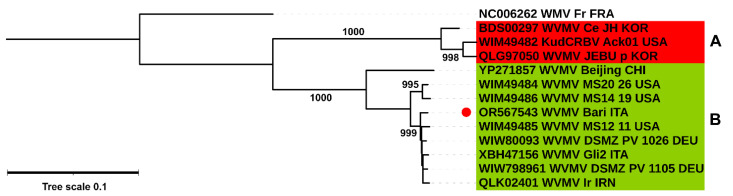
Rectangular phylogram based on the complete sequence of the polyprotein (3092–3095 aa) of eleven isolates of Wisteria vein mosaic virus (WVMV) and one isolate of kudzu chlorotic ring blotch virus (KudCRBV). Phylogenetic reconstruction, based on the maximum Likelihood (ML) approach, was performed with PhyML/SMS workflow available at the NGPhylogeny web service. Tree editing was carried out with Interactive Tree of Life (iTOL) software, version 6.5.8. Bootstrap branch support was computed over 1000 replicates. Bootstrap values above 500 are shown above the branches. The branch length is scaled by evolutionary distance (substitution/site). The tree was rooted using an isolate of watermelon mosaic virus (WMV) as an outgroup. Two clades (lettered A and B, respectively) were found, based on a cluster analysis performed with the TreeClus algorithm [51], with a dissimilarity threshold >50%. The red dot indicates the WVMV isolate Bari, the object of this study. The scale bar represents the branch length values. All sequences are identified by their accession number, virus and isolate names and country of origin. Three-letter codes are used for country names, as defined in the ISO 3166-1 standard [94]. Detailed information on individual isolates can be found in Table S1.

TreeClus analysis revealed the presence of two distinct and well-supported clades (Figure 4). The larger clade (Figure 4, clade B) included all WVMV sequences isolated from *Wisteria* spp. The smaller clade (Figure 4, clade A) grouped isolates identified in non-wisteria hosts, being Ce-JH and JEBU-p, found in jack bean (*Canavalia ensiformis*) and soybean (*Glycine max*), respectively [7,13], and KudCRBV isolated in the US from the leguminous weed kudzu (*Pueraria montana* var. *lobata*). As for WVMV Bari, it was placed in the major clade B, in a sub-clade that included American, Asian and European isolates, regardless of their geographical origin. It could be observed that this subgroup was closely related, but distinct from the reference isolate WVMV Beijing.

### 3.5. Recombination Analysis

The recombination analysis conducted using the different algorithms implemented in RDP4 identified a potential recombination event in the P3 region of WVMV JEBU-P (nt positions 2032–2302, relative to the CDS region). The putative recombination event was statistically significant for six of the nine algorithms tested (RDP: *p*-value 8.71 × 10^−6^; GENECONV: *p*-value 9.18 × 10^−3^; Bootscan: *p*-value 6.92 × 10^−5^; Maxchi: *p*-value 1.55 × 10^−2^; Chimaera: *p*-value 4.46 × 10^−4^; 3Seq: *p*-value 2.99 × 10^−5^) (Table S6).

Based on this prediction, WVMV JEBU-p would have resulted from recombination between the major parent KudCRBV Auck01 and the minor parent WVMV Ce-JH. However, no significant recombination events were found involving WVMV isolates from wisteria hosts.

### 3.6. Dating Analysis

To investigate the divergence time of the available WVMV isolates, a time tree was inferred by applying the RTDT relaxed clock algebraic method to a phylogenetic tree based on the complete CDS and calculated using the ML method. The General Time Reversible (GTR) model [95] with (+*Γ*+I) decoration was selected as the most adequate by SMS prediction (AIC score 78,303.184). The time tree was computed using sampling tip dates available for the eleven isolates of WVMV and KudCRBV Ack01 and used as calibration constraints. The WMV isolate Fr was used as an outgroup, although times were not estimated for outgroup nodes because the RTDT method uses evolutionary rates from the ingroup to calculate divergence times and does not assume that evolutionary rates in the ingroup clade apply to the outgroup.

**Figure 5:**
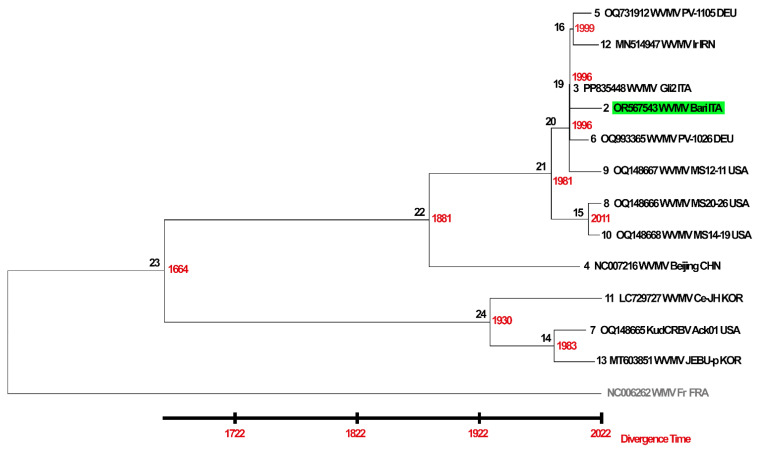
Time tree inferred by applying the Relative Time with Dated Tips (RTDT) method [53] to a phylogenetic tree calculated using the Maximum Likelihood (ML) method and the General Time Reversible (GTR) substitution model with (+*Γ*+I) decoration. The time tree was computed using sampling tip dates for eleven isolates of Wisteria vein mosaic virus (WVMV) and one isolate of kudzu chlorotic ring blotch virus (KudCRBV) and based on their full-length coding sequence (CDS). The watermelon mosaic virus (WMV) isolate Fr was used as an outgroup. Branch support was computed with the Shimodaira–Hasegawa (SH)-like likelihood ratio test. The scale bar represents the divergence time (CE, Common Era, in red). Phylogenetic analysis was conducted using PhyML-SMS version 3.3 and time tree estimation was computed in Mega11. The black numbers in the plot represent the node IDs. The numerical outputs of the analysis are detailed in Table S7. All sequences are identified by their accession number, species and isolate names and country of origin. Three-letter codes are used for country names, as defined in the ISO 3166-1 standard [94]. Detailed information on individual isolates is available in Table S1. The isolate object of this study, WVMV Bari, is highlighted in green.

Based on the tree obtained, the existence of a phylogenetic separation between the isolates affecting wisteria hosts from those found in other species was confirmed. Our molecular clock estimate dated the occurrence of this separation to 1664 Common Era (CE), although a 95.66% confidence interval (CI) widened this range, placing the date between 1454 and 1792 CE (Figure 5, Table S7). Interestingly, the first subsequent differentiation would have occurred within the ‘wisteria clade’, leading around the 1880s (1791–1930 CE, CI 95.81%) to the evolution of the group most numerous at present. This subclade, of heterogeneous geographical origin, would have evolved distinctly from the pathway that led to the emergence of the isolate Beijing, until now considered the reference for the species.

A few years later, virus evolution would have continued, leading to genetic differentiation between the isolates affecting non-wisteria hosts. The RTDT prediction dated the node leading to the emergence of WVMV Ce-JH to 1930 CE (1877–1961, CI 95.81%), and the subsequent differentiation between the closely related KudCRBV Ack01 and WVMV JEBU-p to 1983 CE (1952–1996, CI 95.81%).

A differentiation of minor entity, and therefore more condensed in time, would have later occurred among the other wisteria variants. The subclade bearing the isolate Bari, the main subject of this study, may have emerged approximately 30 years ago (1964–1995 CE, CI 95.81%), in a process of further differentiation not yet accentuated. This has proceeded so far to the US isolates MS20-26 (OQ148666) and MS14-19 (OQ148668), presumed to belong to the most recently emerged subclade (1986–2017 CE, CI 95.81%).

### 3.7. Network Inference Analysis

**Figure 6:**
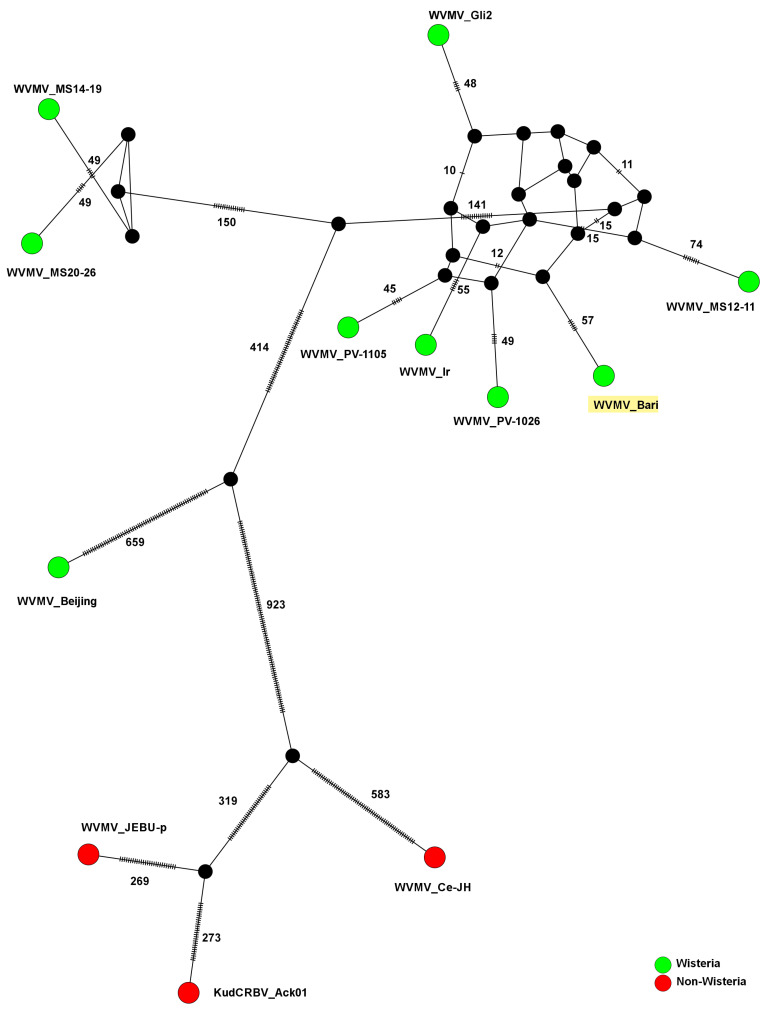
Network inference analysis conducted on the full-length coding sequence (CDS) of eleven isolates of Wisteria vein mosaic virus (WVMV) and one isolate of kudzu chlorotic ring blotch virus (KudCRBV). The labelled circles represent the sequenced isolates, coloured according to the host plant (green: *Wisteria* spp.; red: non-*Wisteria* spp.). The median vectors shown as unlabelled black dots represent the hypothetical intermediate variants. The hatch marks and the numbers indicate the mutations separating the variants (one hatch mark equates to ten mutations). Mutation numbers below 10 are not represented in the plot. The Integer Neighbour-Joining (NJ) network was created using the PopART (Population Analysis with Reticulate Trees) software version 1.7 (Leigh and Bryant, 2015) and selecting the ‘Integer Neighbour-Joining Net’ option, with default settings. All sequences are identified by their virus acronyms and isolate names. Detailed information on individual isolates is available in Table S1. The isolate object of this study, WVMV Bari, is highlighted in yellow.

To better investigate the evolutionary pathways that led to the genetic differentiation between the hitherto sequenced isolates of WVMV and KudCRBV, an integer NJ network was constructed based on the CDS region. This analysis resolved (with higher accuracy) the evolutionary links that led to the emergence of the different variants of the species, or two different species when considering the closely related KudCRBV. The network that was built on the few available sequences not only grouped the three divergent isolates WVMV Ce-JH, WVMV JEBU-p and KudCRBV Ack01, but also better evidenced a distinction between the isolates infecting wisteria hosts (Figure 6).

Based on this analysis, there could be at least two hypothetical missing intermediates (black circles in Figure 6) totalling 659 + 923 mutations, which separate the clade of non-wisteria hosts from the reference isolate Beijing. In this context, the pathway leading to the emergence of WVMV Bari appeared much more complex. The clade grouping WVMV Bari with PV-1105, PV-1026, Ir and MS12-11 would be evolutionarily distant from the first point of divergence with non-wisteria isolates. The distance between this clade and the other Italian isolate, WVMV Gli2, was better highlighted than by relying on sequence identities alone. In addition, a high number of hypothetical variants lacking in current knowledge was predicted by the occurrence of the intricate reticulum of median vectors shown in the plot. Two out of the three American isolates (WVMV MS20-26 and MS14-19), those assumed to be of more recent emergence from the dating analysis, would be following a delineated differentiation that clearly separated them from the largest cluster.

### 3.8. Analysis of Genetic Differentiation and Population Dynamics

An analysis of the genetic differentiation and population dynamics potentially existing between the two main groups identified in previous phylogenetic reconstruction (Figure 4) was attempted. To this end, the available viral sequences were grouped into two sub-populations according to the host species of origin. Comparison based on the full-length CDS was therefore established between the phylogroup containing isolates from *Wisteria* spp. (n = 9) and that comprising isolates from non-wisteria hosts (n = 3).

The genetic differentiation between the two populations was calculated through the fixation index (*F*_ST_) and was found to be 0.67. This value showed a good degree of genetic differentiation, on a scale with 0 (undifferentiated populations) and 1 (fully differentiated populations) as extreme reference values [96,97]. The *F*_ST_ index > 0.33 also suggested an infrequent gene flow between the two phylogroups. This evidence was also confirmed by the low value of the number of migrants per generation (*N*m) being 0.13 [98] (Table 1).

The existence of a relevant degree of genetic differentiation was further evidenced by the high values resulting from the computation of other inter-population parameters: the average number of nucleotide substitutions per site between populations (*D*_xy_, 0.233), the average proportion of nucleotide differences between populations (*K*_xy_, 2157.185) and the number of net nucleotide substitutions per site (*D*_a_, 0.15). The results were further validated with *K*_s_*, Z* and *S*_nn_ statistical permutation tests and all were significant, confirming the reliability of the prediction and the occurrence of a process of genetic differentiation between the two groups (Table 1).

Neutrality tests were used to estimate the hypothesis of neutral selection operating on the polyprotein gene in the wisteria-related group (Table 2). The negative values found in Tajima’s *D* (−1.283), Fu and Li’s *D* (−1.306) and Fu and Li’s *F* (−1.463) tests may disclose a low-frequency polymorphism, therefore related to an excess of rare alleles. This trait well describes a population undergoing a phase of expansion or purifying selection. However, none of the tests returned statistically significant values, so it was not possible to determine, based on the few available sequences, whether population selection is evolving under non-random processes. The statistical evaluation of the neutrality hypothesis could not be performed for the non-wisteria host group due to the limited number of isolates so far available, still below the minimum required (n = 4).

When the possible action of selective pressure was investigated, it was found that the d*N*/d*S* ratio between the average number of synonymous and non-synonymous substitution per site was well below 1 in both populations (wisteria isolates: 0.074, non-wisteria isolates: 0.025) (Table 2). This may suggest that in both phylogroups a negative purifying selection is acting on the polyprotein gene.

To further investigate if these selection forces could act at a site-specific level, a SLAC analysis was performed on the taxonomically relevant CP cistron, also relying on a slightly larger number of sequences available. The SLAC algorithm, with the *p*-value threshold set at 0.1, found evidence of pervasive negative/purifying selection at 28 codon sites, evenly distributed along the CP sequence, at codon positions 36, 65, 68, 80, 81, 87, 88, 95, 96, 100, 112, 117, 151, 161, 174, 178, 183, 190, 207, 209, 219, 220, 222, 223, 253, 266, 267 and 271 (Table S8, Figure S5). No evidence of sites under positive/diversifying selection was found.

## 4. Discussion

Naturally occurring with a large number of species (228 member species and 28 tentative unclassified species) and a host range comprising approximately sixty plant families, the genus *Potyvirus* is increasingly considered a good candidate for evolution studies [99]. The most recent phylogenetic reconstructions have suggested that continuous host gains have significantly influenced the genetic diversification of potyviruses, rendering the evolution of this genus very dynamic.

This is even more evident in the largest clades, characterised by an older history and a wider host range, as in the case of the “BCMV supergroup” [91]. This widely studied group is, together with that of the potato virus Y (PVY), one of the largest lineages in the genus *Potyvirus* and includes more than 35 known species [90]. This group has attracted the attention of researchers mainly because of the significant damage some species have caused to primary crops in different regions of the world, as in the case of BCMV to the common bean (*Phaseolus vulgaris*), WMV and zucchini yellow mosaic virus (ZYMV, *Potyvirus cucurbitaflavitesselati*) to cucurbits, soybean mosaic virus (SMV, *Potyvirus glycitessellati*) to soybean and cowpea aphid-borne mosaic virus (CabMV, *Potyvirus vignae*) to cowpea (*Vigna unguiculata*) and passion fruit (*Passiflora edulis*). However, BCMV group species are also showing increasing cosmopolitan distribution and the ability to cause serious disease in numerous minor crops, ornamental species and invasive wild plants or endemisms [90].

Among the BCMV group members, WVMV has been gaining increasing popularity in recent years. The associated disease, WMD, can significantly impair the visual appeal of wisterias and deplete their commercial value. Therefore, WVMV is regarded with increasing concern by nursery producers worldwide. Before 2023, almost all available WVMV reports were based on the detection of the virus by RT-PCR and sequencing of the corresponding amplicons, whereby the genomes of only four isolates had been completely sequenced. It can be seen that since our previous report [7], the first to provide molecular evidence of the presence of WVMV in Italy, a collective effort over the past two years has significantly increased the data available on this virus. The genome sequences of three WVMV isolates found in Mississippi (MS20-26, OQ148666; MS12-11, OQ148667; MS14-19, OQ148668) [5] have been made available in GenBank, along with those of the two German isolates PV-1105 (OQ731912) PV-1026 (OQ993365) and the Italian isolate Gli2 (PP835448), retrieved from the DSMZ [100] and EVAglobal [101] collections, respectively.

To collaborate in this effort and further corroborate the preliminary data gathered in our previous investigation, in the present study the complete sequence of a WVMV isolate collected from *W. sinensis* in Apulia, southern Italy, was obtained. As expected, the genomic structure of WVMV Bari (OR567543) exactly matched that of the other isolates known in the species. The proteolytic processing sites in the WVMV Bari polyprotein were mostly conserved. However, it is interesting to note the occurrence of low-frequency amino acids, as in the case of the isoleucine found in 6K1/CI and CI/6K2 sites. These apparent violations of the consensus sequences described so far for potyvirus cleavage sites may prompt future studies aimed at analysing cleavage sites of multiple sequences from the same species, so that new consensus sequences could be defined at the species level.

Since the HTS output returned a very high coverage, it was possible to conduct a variant call analysis of the assembled sequence. This approach is increasingly used in NGS studies conducted on RNA viruses [102] because it is very common for these viral species to be present in a single plant with multiple variants. These biological entities are referred to as quasispecies [103]. In the case of WVMV Bari, the presence of a fairly high number of single nucleotide variants, albeit with an average frequency of less than 10%, would suggest the existence of quasispecies. This would not come unexpected, as it is consistent with the evidence that potyviruses have large mutation rates and frequently form quasispecies populations [104]. The fact that the highest number of potential SNVs were predicted in the NIb region, and for the most part attributable to non-synonymous amino acid changes, is intriguing. NIb is considered one of the most constrained proteins among those encoded by several potyviruses [105], and non-synonymous changes are expected to be uncommon as they may induce fitness penalties for the species [106].

A main effort of this work was to reconstruct the still unclear phylogenetic and evolutionary relationships of WVMV Bari and the other isolates of the species. Phylogenetic reconstruction based on the available potyvirus polyprotein dataset confirmed the placement of WVMV within the BCMV supergroup, very close to other species. These include a virus of uncertain classification, recently discovered by Aboughanem-Sabanadzovic et al. [5] and provisionally named KudCRBV. *P. montana*, the host plant of this virus, usually reported under the vernacular name of ‘kudzu’, is, like wisteria, another member of the Fabaceae family, also considered native to South Asia.

KudCRBV has been isolated in Mississippi, where kudzu is considered (as in many countries) a dangerous and widespread invasive weed. We found members of other representative species isolated from Fabaceae hosts in the same clade of WVMV, namely SMV from soybean and UMV originally isolated in Taiwan [107] from the ornamental shrub *Uraria crinita*. The taxonomic closeness of SMV with WVMV is of particular relevance, as SMV represents a widely recognised major threat, and this sublineage is sometimes cited as the ‘SMV cluster’ of the BCMV group. Apart from those infecting Fabaceae, other viruses found to be closely related to WVMV were WMV, also considered a major threat to Cucurbitaceae hosts, and CLLV, responsible for latent infections in calla lily plants (*Zantedeschia aethiopica*).

Besides outlining the placement of WVMV in the context of potyvirus diversity, the present work has provided the most interesting insights into the peculiarities of its interspecific diversity.

Based on phylogenetic and pairwise comparisons, all known isolates of WVMV (for which the coding region was available) could be grouped into two clearly distinguishable lineages. Bringing further confirmation to what has already been observed by Aboughanem-Sabanadzovic et al. [5] on a smaller number of isolates, there was a clear division between isolates found in wisteria species from those that could be defined as the ‘non-wisteria clade’. KudCRBV fitted neatly into the second cluster, together with the two WVMV isolates JEBUp (soybean) and Ce-JH (jack bean).

Pairwise analysis well supported this clear-cut clustering between the two clades, helping to highlight the doubts recently raised about the correct taxonomic attribution of KudCRBV and the divergent isolates of WVMV. The two clades share sufficiently high intra-group sequence identities not to question their belonging to the same species. However, the same cannot be said when comparing inter-group similarity rates. If one refers to the commonly adopted inter-species demarcation thresholds for potyviruses, these are generally accepted as <76% nucleotide identity and <82% amino acid identity, based upon the large ORF or its protein product [108]. It is relevant, therefore, to note that the percentages of identity with the RefSeq WVMV Beijing come very close to these demarcation thresholds, not only for KudCRBV (76.8% nt, 82.6% aa), but likewise for WVMV Ce-JH (76.6% nt, 83.0% aa) and JEBU-p (77.1% nt, 82.6% aa). This borderline taxonomic placement appears even more relevant, given that these low similarity values with WVMV Beijing are very similar to those with WMV Fr (76.5% nt, 81.3% aa) and SMV N (76.5% nt, 81.0% aa). The latter are, indeed, considered the reference sequences of the two respective species, well recognised and characterised as distinct taxonomic entities.

P1-Pro is often mentioned as the least conserved region within the potyvirus polyprotein as it shows strikingly high variability, especially in its N-terminal portion [73]. These traits were also confirmed in our observations. Interestingly, it was found that high sequence divergences, up to 70% when looking at the first 100 N-terminal amino acids, separate the wisteria from the non-wisteria isolates. It has been suggested that this hypervariable region in P1-Pro may have a prominent role in the evolutionary diversification of potyviruses and may be relevant for host–virus interaction and, thus, host range definition [109,110,111,112]. It is worth mentioning that in the very N-terminal P1-Pro region of the soybean-infecting isolate WVMV JEBU-p we could identify two distinct motifs, _12_SxPxTH_17_ and _31_NSV_33_, respectively, which are perfectly conserved in SMV N, but also in WVMV Ce-JH and KudCRBV Ack01 and are not present in any of the isolates of the wisteria-infecting clade. These findings could support the role that P1-Pro (and particularly its N-terminus) may have played in the adaption to different hosts of the non-wisteria clade.

As already pointed out by Aboughanem-Sabanadzovic et al. [5], the taxonomic placement of WVMV, KudCRBV and these borderline isolates is currently an open question, which is deferred for further discussion. The demarcation criterion based solely on sequence identity could indeed be considered a non-exhaustive simplification in some cases. It is equally true that highly divergent strains could be the result of adaptations to hosts outside the known conventional range for the species. However, it is also easy to consider that the very clear distinction between the isolates identified in wisteria (the host of choice for the WVMV species) and those found in other Fabaceae points to a different taxonomic attribution in which the host is the diriment biological trait. The need for taxonomic reallocation has arisen with some frequency in recent years within the potyviruses and precisely in species that are not phylogenetically distant from WVMV. In this regard, the recent cases of UMV originally classified as a divergent strain of SMV [113], and the virus causing passion fruit woodiness disease in Taiwan, for years regarded as passion fruit woodiness virus (PWV) but now reclassified as a strain of East Asian Passiflora virus (EAPV, *Potyvirus orionspassiflorae*), should be mentioned [114].

Recombination has often been considered a relevant process in the biological evolution of the *Potyvirus* genus [115,116] and is often mediating adaptive changes to new hosts and the emergence of novel epidemics [117]. In the case of WVMV, no significant traces of recombination events were found in isolates infecting wisteria. However, a recombination signal involving isolates of the divergent clade was instead predicted within the P3 region. If confirmed, a remodelling in that region of the polyprotein might have interfered in some way with the determination of the host range [118]. It must be considered, however, that the presence of recombination events in any of the two clades may find more confirmation, or conversely be disproved, once more sequences become available.

The involvement of KudCRBV in this recombination pattern, presumably as a major parent, might suggest that the three strains or this putative species may share a recombination history, starting from a common ancestor shared with the species infecting wisteria hosts. The information available at present appears to be far from robust evidence. However, recombination in this clade may have occurred to increase adaptive fitness to the new host range [119,120].

Phylogeographic analyses of the speciation pattern of the BCMV group suggested that this clade of potyviruses originated in the Far East, before some species migrated to Oceania and others to the West. Based on the estimations of Gibbs and Oshima [90], the occurrence of the divergence in potyvirus evolution that led to the clustering of the BCMV group would be dated to no less than around 3000 years ago. In reconstructing the evolutionary history of this intriguing subgeneric group, molecular clock data often have to be combined with the domestication history of host plants. In this sense, it is plausible to speculate that the earliest virus–host encounters may have occurred in crops that were historically rooted in certain territories. As such, it is conceivable that the common bean only became a BCMV host after its more recent domestication in the American continent, even though the virus has been present in crops originating in the Asian continent for a longer time.

Conversely, the relationship between CAbMV and a long-established Southeast Asian species such as cowpea (*Vigna* spp.) is presumably much older. The same can be said for SMV, now considered pandemic, but with a very long evolutionary history parallel to that of its host of choice (soybean), domesticated over three thousand years ago by *Glycine* spp. and indigenous to East Asia [115]. Similarly, other species of the BCMV group which are widespread today, such as WMV and ZYMV, have changed their host range over time. Today, the two viruses constitute a major issue for *Cucurbita* spp. However, it is safe to assume that before the domestication of these plants in the American continent, WMV and ZYMV infected cucurbits originating in the African and Asian continents, i.e., watermelon, melon and cucumber, among others, for tens of centuries [90,121].

As for WVMV, our dating attempt places the occurrence of separation between wisteria and non-wisteria infecting isolates in a time interval centred around the second half of the 17th century. The finding corroborates current knowledge that places the origin of WVMV in Eastern Asia, which is also the place of origin and greatest natural spread of the species *W. sinensis*, widely distributed in northern, central and southern China, *W. floribunda*, common in Japan and Korea and *W. venusta*, endemic to Japan [122]. The Eastern Asian origin of the virus has also recently been hypothesised on the basis of the highest indices of genetic diversity measured in Asian isolates. The assumption is, in fact, that the centres of origin of a pathogen are usually also related to higher genetic diversity [123].

Our time tree analysis found that the subsequent radiation between the different isolates infecting *Wisteria* spp. would have started from the end of the 19th century onwards, in a process of slow but progressive differentiation from the isolates of origin, probably typified by the Chinese isolate Beijing. The most plausible hypothesis for the subsequent worldwide propagation of the virus is linked to trade mediated by human activities, which became increasingly intense from the 20th century onwards. Few data are currently available to fill the knowledge gap on the evolution of the clade infecting non-wisteria hosts. Our preliminary reconstruction places, however, the differentiation between the few known isolates at a later time than that between the isolates of the wisteria branch. This delayed occurrence may provide support for the more plausible hypothesis suggesting the origin of a new species, or of highly divergent strains from the original WVMV, as an adaptation to new hosts in the Fabaceae; not necessarily domesticated, but certainly coexisting in the Southeast Asian cradle of origin.

The process of speciation (completed or still ongoing—this remains to be clarified) may also have benefited from recombination events, as reported above. The ease of propagation of WVMV in herbaceous hosts of numerous families and the recent evidence gathered, at least under experimental conditions, on the efficacy of its aphid-mediated transmission makes this scenario quite plausible. This viral trafficking between cultivated, non-crop and wild plant species seems to be a constant feature that, as mentioned above, has made the evolution of the BCMV potyvirus supergroup lively. The earlier evolution of WVMV itself may have followed the same pattern, and it is conceivable that at least the dawning stages of natural evolution between wisteria and non-wisteria hosts may have unfolded in this way. Clearly, and subsequently, human intervention did the rest. There is an intense trade in ornamental plants between Europe, the USA and Oceania and the territories of origin and subsequent establishment of the different wisteria species. Not to mention that since the first introduction of *W. sinensis* and *W. floribunda* to the North American continent, the two species have become invasive and their spread has often crossed even natural area boundaries [124].

A similar pattern is featured in the spread of kudzu. Although much less known worldwide, this plant, also of Southeast Asian origin, reached the US in the late 1800s promoted as an ornamental shading plant and then to cope with soil erosion. However, over time it turned out to be an invasive species—which, unlike wisteria, has a very negative reputation for significant ecological impact, apart from the viruses it can host [125]. Easily, the wide geographical spread of WVMV and the discovery of a possible new related species of Asian origin in the American continent are strongly correlated with trade-mediated circulation patterns and the subsequent ecological establishment of their hosts. This aspect may sound quite alarming, as these ‘new-encounter scenarios’ between potyviruses and crop (as well as non-crop hosts) from another territory have often resulted in new, even severe, diseases [17,116].

The clear separation existing between WVMV isolates and those putatively assigned to the KudCRBV clade is reinforced by network inference analysis. This computational approach permits even visual resolution of the network relationships existing between the biological entities of interest, and is particularly useful in datasets that lack experimental data on their interactions [126]. Mainly used in the context of prokaryotes, it has more recently (in the post-pandemic era) proved useful for viruses. In our case, it has been particularly helpful in substantiating the evolutionary paths of the WVMV/KudCRBV isolates. Remarkably, we are far from a full understanding of what occurred in this speciation process, as many intermediate variants could be predicted but are still unknown. A greater complexity has been observed in the portion of the network that encompasses WVMV Bari and all other isolates that have differentiated more recently as a result of the virus’ geographical spread across continents.

This greater complexity is revealed with a highly connected network that is indicative of a slower evolutionary speed [127], as one would expect from a differentiation that appeared more recently and is still not very pronounced, barely referring to sequence identities. However, as mentioned above, the presence of a large number of hypothetical intermediate variants, present in the network in the form of median connection vectors, warns of the possibility that evolutionary distances may be wider and more complex than sequence identities show. Clear divergences emerge in the topology of the network under investigation, with intermediate relationships yet to be discovered even between isolates collected in the same country of origin. This is the case with WVMV Bari and the other Italian isolate Gli2, or the American isolates MS20-26 and MS14-19, placed far apart in a pattern clearly distinct from MS12-11. This evidence reinforces the hypothesis that the circulation of propagation material may have played a greater role than vector-mediated transmission [7,12,14].

The analysis of population genetics parameters provided further evidence of the role of the host in differentiating into two ‘sub-populations’, distinguishable on the basis of their genetic structure, the isolates infecting wisteria, from those of the ‘non-wisteria clade’.

Based on the high value of the fixation index (*F*_ST_), it could be estimated that the two phylogroups currently represent two genetically distinct populations and seem to have almost completed their process of genetic differentiation. These considerations were well supported by the values observed in all inter-population genetic differentiation parameters. Notably, the average inter-population nucleotide difference (*K*_xy_) was found to be very high, thus suggesting a long-established separation between the two groups and restriction in gene flow [128]. The combination of the absolute value of *F*_ST_, which crossed the threshold value of 0.33, and the low number of migrants per generation (*N*_m_), well below 1, substantiates the relative absence of current gene flow between the two populations according to several authors [98,129]. In the absence of a dynamic gene flow, the scenario again points to a stable genetic divergence between the two phylogenetic groups. The availability of more sequences will better corroborate this hypothesis.

However, the statistical significance of three permutation tests widely used for validation (*K*_s_*, Z* and *S*_nn_) makes this prediction reasonably reliable [63]. In the last decades, a plethora of studies have related low gene flow amount to the “barrier” concept, and a wide variety of modes of action have been proposed to explain their effects on genetic differentiation [130]. Besides geographical constraints, several mechanisms may impede or interfere with gene flow, and the presence of biological or molecular barriers has also been proposed in relation to RNA plant viruses. Once the presence of highly structured populations was confirmed, it would be interesting to investigate whether competition between minor and well-established host-bound variants could act as a barrier and limit or reshape gene flow between WVMV-related strains. Interestingly, this mechanism has been proposed to explain the dynamics of emerging WMV populations in France [131].

Tajima’s *D*, Fu and Li’s *D* and Fu and Li’s *F* tests applied to assess the selective neutrality of the nucleotide variety (limited to the wisteria-associated population) resulted as negative, although not statistically significant. Similarly, negative and statistically insignificant neutrality tests were reported for other potyviruses in populations of turnip mosaic virus (TuMV, *Potyvirus rapae*) [129], EAPV and peanut mottle virus (PeMoV, *Potyvirus arachidis*) [115] as well as in other genera, as in the case of Southern tomato virus (STV, *Amalgavirus lycopersici*) [96,132] or grapevine Pinot gris virus (GPGV, *Trichovirus pinovitis*) [97]. Positive Tajima’s *D* values could be expected if a population was the result of a recent admixture [133] and this is consequently not the case for the WVMV wisteria group. Otherwise, the negative values found could explain an expansion of the population as a consequence of a bottleneck or a selective sweep [129,134]. In this situation, an excess of low-frequency polymorphisms, so-called ‘rare alleles’, is often measured, and may even be the result of spillover [84,115].

A low-frequency polymorphism is often indicative of the action of purifying selection [135]. When the evolutionary selection pressure acting on the coding region in both host-associated virus sub-populations was evaluated, the dN/dS ratio being < 1 indicated that both groups are under negative selection or stabilizing evolutionary constraints [96].

This purifying selection force is possibly lessening the variability in both populations [134], intending to remove isolates with potentially deleterious mutations and reduce the frequency of viral variants with lower selective fitness advantage [136,137]. As a result, negative selection fosters a high stability of the virus genetic structure. This condition, in turn, may suggest that WVMV strains are currently well-adapted in their environment [12,138]. This scenario suggests a long-standing co-evolution between the virus and its hosts [139] and indirectly confirms the outputs of the dating analysis. Overall, the occurrence of strong evidence of negative selection perfectly matches what has been reported in numerous previous studies on potyviruses [115].

The CP region has a remarkable role in potyvirus pathogenicity and may harbour host-related determinants that may differ between strains in some species. Its biological and functional peculiarities make this genome region a potential target for selection forces acting at both plant and vector stages [140]. In the case of WVMV, relying on the SLAC codon-based ML method, evidence was found of purifying selection codon sites evenly scattered along the CP region, thus confirming preliminary investigations [7].

In their extensive investigation, at the genus scale Nigam et al. found that negative-selection sites in the potyvirus genome are largely predominant over those which undergo diversifying selection, which makes the coding sequence markedly stable. The CP region does not escape this logic [83]. However, its N-terminal portion is often a hypervariable region, having a multifunctional role and being involved in both vector transmission and systemic colonisation of the plant, which may consequently exhibit sites under positive selection [140,141]. Nevertheless, no evidence of codon sites under positive selection was found in the sequences analysed in this study.

However, as reported in several potyvirus species including PVY, bean yellow mosaic virus (BYMV, *Potyvirus phaseoluteum*), yam mosaic virus (YMV, *Potyvirus yamtesselati*) and plum pox virus (PPV, *Potyvirus plumpoxi*), among others [142], the CP gene remains an interesting candidate for future studies on the evolution of WVMV and its host adaptation.

The possible evolutionary pattern that emerges from the analysis of WVMV and possibly allied species raises intriguing questions about the potential of this virus to increase its danger. Several factors, as seen above, have contributed and may further contribute to its spread. As mentioned, the evolution of potyviruses and their speciation mechanisms seem to be strongly influenced by agricultural changes [99]. But equally remarkable is the possibility that the ability to switch hosts and adaptation, which seems to have characterised the early stages of genetic differentiation within the WVMV species, may contribute to its further spread even outside the currently known host limits. The ability of the virus to infect a wide assortment of indicator plants belonging to numerous botanical families has been extensively proven under experimental conditions [2].

The finding that WVMV may have evolved into strains or species able to successfully adapt to economically important crop hosts such as soybean and jack bean, as well as non-domesticated species with high invasive potential such as kudzu, chimes with alarm. The taxonomic closeness of SMV, which presumably shares the same geographical origin and has caused devastating effects precisely in soybean, demonstrates how host jumping, deemed a cornerstone in the evolution of pathogens [143], must be considered with great regard in this species cluster.

Valouzi et al. [14] identified WVMV in black bean (*Aphis fabae*) and cowpea (*A. craccivora*) aphids feeding on WVMV-free *Robinia* spp. trees. Under controlled conditions, both species transmitted the virus to bean seedlings. Similarly, Aboughanem-Sabanadzovic et al. [5] proved that potato (*Macrosiphum euphorbiae*) and cotton (*A. gossypii*) aphids can artificially vector KudCRBV to kudzu, and the latter also to soybean. The ability of the virus to be transmitted via aphids could make viral trafficking to and from wild or cultivated herbaceous Fabaceae relatively easy. The potential spread of WVMV in additional and as yet unknown reservoir species, or further unpredictable spillover, makes its ecological risk even greater.

Furthermore, when present in a severe state, WMD symptoms significantly compromise the marketing of wisteria plants and the virus can already constitute a major concern for the nursery sector, especially where ornamental plants are highly valued, as in Italy [12].

## 5. Conclusions

The present work, providing the complete genome sequence of the WVMV isolate Bari, completes the study initiated in our previous effort. Our findings, together with data recently collected by other research groups, bring further and definitive confirmation of the presence of WVMV in Italy associated with WMD, as hypothesised in the 1970s.

There is growing evidence that the virus, most likely originating in the Far East (the place of origin of wisteria, its main known host), is spreading to Europe and worldwide.

Well-supported phylogenetic analyses revealed a clear-cut divergence of known WVMV isolates into two distinct phylogroups, separated on the basis of their hosts (i.e., wisteria vs. non-wisteria lineages). The non-wisteria clade also includes a putatively assigned new species (KudCRBV) and the whole lineage now represents an intriguing borderline case for taxonomic assignment. Recombination events and higher genetic divergence of the non-wisteria clade could be a consequence of host adaptation. RTDT dating analysis traces back the origin of WVMV and its evolutive divergence to the 17th century. The wisteria clade may have originated and begun its (still ongoing) slower differentiation even earlier.

Whether this process of speciation has been completed or is still in progress remains to be clarified, although the evidence we have gathered, based on the available data, suggests the former hypothesis. What seems relevant is that the evolution of different strains of the same species (or two distinct species) in the main hypothesis may be the result of viral traffic between domesticated and non-domesticated plant species, subsequently also mediated by human action. These spillover mechanisms, already widely hypothesised in the *Potyvirus* genus, provide an important alarm bell for the future spread of WVMV and new emerging threats.

We wish to emphasise that all the information we have gathered, and the survey methodologies employed, are intended to contribute to the proposition of a case study that can direct subsequent studies towards an evolutionary approach to explain the emergence of potyviruses and other plant viruses in general. This will allow closer monitoring and a deeper understanding of the patterns of emergence of new threats, which are to be expected in an environment increasingly subject to change and to the effects of globalisation.

## Figures and Tables

**Table 1:** Indices of genetic differentiation and gene flow between host-related populations of wisteria vein mosaic virus (WVMV) and kudzu chlorotic ring blotch virus (KudCRBV) isolates, based on their full-length coding sequences.

Population A	Population B	*F*_ST_	*N*m	*K*xy	*D*xy	*D*a	*K*_s_*	Z*	*S*_nn_
Wisteria hosts (n = 9)	Non-wisteria hosts (n = 3)	0.67	0.13	2157.185	0.232	0.155	5.87 ^††^	2.77 ^††^	1.00 ^†^

**n:** number of isolates; ***F*_ST_:** Wright’s *F* fixation index; ***N*m:** number of migrants per generation, [(1/F_ST_) − 1]/4; ***K*xy:** average proportion of nucleotide differences between populations; ***D*xy:** average number of nucleotide substitutions per site between populations; ***D*a:** number of net nucleotide substitutions per site between populations. *K*_s_*, Z* and *S*_nn_ statistical tests [62,63] were performed with 0.05 α significance level (^††^: *p* < 0.01; ^†^: *p* < 0.05). All genetic measures were computed on DNA Sequence Polymorphism (DnaSP) software version 6.12.03.

**Table 2:** Evaluation of selection pressure and tests of neutrality of wisteria vein mosaic virus (WVMV) and kudzu chlorotic ring blotch virus (KudCRBV) isolates, grouped by host origin, and based on their full-length coding sequences.

Population	n	d*N*	d*S*	ω	Tajima’s *D*	Fu-Li’s *F*	Fu-Li’s *D*
Wisteria hosts	9	0.016	0.209	0.074	−1.284 ^ns^	−1.463 ^ns^	−1.306 ^ns^
Non-wisteria hosts	3	0.016	0.623	0.025	n.a.	n.a.	n.a.

**n:** number of isolates; **d*N*:** average number of non-synonymous mutations per non-synonymous site; **d*S*:** average number of synonymous mutations per synonymous site; **ω:** d*N*/d*S*, average ratio between non-synonymous and synonymous mutations in pairwise sequences; **ns:** not significant at 0.1 *α* level (*p*-value > 0.1); **n.a.:** not available, due to limited number of isolates available. All genetic measures and statistical tests were computed on DNA Sequence Polymorphism (DnaSP) software version 6.12.03.

## Data Availability

Nucleic acid sequence data relative to the WVMV isolate Bari have been deposited in GenBank under the accession number OR567543. Raw high-throughput sequencing data have been deposited in the NCBI Sequence Read Archive (SRA) repository (BioProject PRJNA1177767). Any other raw dataset generated and/or analysed for the aim of the current study is available from the corresponding author upon reasonable request.

## Supplementary Materials

The following supporting information can be downloaded at: https://www.mdpi.com/article/10.3390/pathogens13111001/s1, Table S1: List of virus sequences used in this study; Table S2: Summary of BLASTX analyses of de novo assembled contigs from reads generated by high-throughput sequencing (HTS) of the *Wisteria sinensis* tree affected by Wisteria mosaic disease (WMD); Table S3: Genomic structure of WVMV isolate Bari with putatively encoded proteins and predicted cleavage sites; Table S4: List of conserved domains common to known potyviruses, identified in the polyprotein of WVMV isolate Bari; Table S5: Single nucleotide variant (SNV) call analysis performed on the complete coding sequence of WVMV isolate Bari; Figure S1: Distance matrix of pairwise MAFFT-based comparison of the complete coding sequence of eleven isolates of WVMV and one isolate of KudCRBV; Figure S2: Distance matrix of pairwise MAFFT-based comparison of the complete sequence coding for the coat protein (CP) of fourteen isolates of WVMV and one isolate of KudCRBV; Figure S3: Distance matrices of pairwise MAFFT-based comparison of the complete or N-terminal sequences coding for the P1-Pro of eleven isolates of WVMV and one isolate of KudCRBV; Figure S4: Distance matrix of pairwise MAFFT-based comparison of the sequence of a 525 nt-long fragment spanning the genes for the nuclear inclusion b (NIb) and capsid proteins of ten isolates of WVMV found in Italy; Table S6: Putative recombination events identified by the detection algorithms of the RDP4 software within the coding sequence (CDS) region of WVMV and KudCRBV isolates; Table S7: Numerical summary of the dating analysis conducted on the coding sequence of WVMV and KudCRBV isolates; Table S8: Codons found under negative selection according to a SLAC analysis conducted on the coding sequence of the coat protein of WVMV and KudCRBV isolates; Figure S5: Plot of the SLAC analysis conducted on the coding sequence of the coat protein of WVMV and KudCRBV isolates.
