# Supplementary material for: The Evolution of Wisteria Vein Mosaic Virus: A Case Study Approach to Track the Emergence of New Potyvirus Threats
Source: Pathogens. 2024 Nov 15;13(11):1001. doi: 10.3390/pathogens13111001 (PMC11597161; doi:10.3390/pathogens13111001)
Supplement: Supplementary file 1 [file pathogens-13-01001-s001.zip › Figure S1.pdf]

Figure S1. Distance matrix of pairwise MAFFT-based comparison of the complete coding sequence (9,279-9,288 nt; 3,092-3,095 aa) of eleven isolates of Wisteria vein mosaic virus (WVMV) and one isolate of kudzu chlorotic ring blotch virus (KudCRBV). The percentage of nt and aa sequence identities are shown below and above the diagonal, respectively. The analysis was performed using Sequence Demarcation Tool (SDT) software, version 1.2. All sequences are identified by their accession number, virus and isolate names, country of origin. Three-letter codes are used for country names, as defined in the ISO 3166-1 standard [94]. The WVMV isolate Bari, the object of this study, is highlighted and its identity values are reported in bold. Detailed information on individual isolates can be found in Table S1.

	**OR567543|WVMV|Bari|ITA**	PP835448.1|WVMV|Gli2|ITA	YP_271857.1|WVMV|Beijing|CHI	WIW79896.1|WVMV|DSMZ_PV1105|DEU	WIW80093.1|WVMV|DSMZ_PV1026|DEU	WIM49482.1|KudCRBV|Ack01|USA	WIM49484.1|WVMV|MS2026|USA	WIM49485.1|WVMV|MS1211|USA	WIM49486.1|WVMV|MS1419|USA	BDS00297.1|WVMV|WVMVCeJH|KOR	QLK02401.1|WVMV|Ir|IRN	QLG97050.1|WVMV|JEBUp|KOR
**OR567543|WVMV|Bari|ITA**		**98.9**	**91.9**	**99.0**	**99.0**	**82.8**	**97.5**	**98.7**	**97.3**	**83.3**	**99.0**	**82.6**
PP835448.1|WVMV|Gli2|ITA	**98.7**		91.9	99.2	99.2	82.8	97.5	99.0	97.3	83.3	99.1	82.6
YP_271857.1|WVMV|Beijing|CHI	**86.1**	86.2		91.8	91.9	82.6	91.9	91.9	92.1	83.0	91.9	82.6
WIW79896.1|WVMV|DSMZ_PV1105|DEU	**98.7**	98.9	86.0		99.3	82.8	97.5	99.0	97.3	83.3	99.2	82.6
WIW80093.1|WVMV|DSMZ_PV1026|DEU	**98.7**	98.8	86.1	98.9		82.8	97.6	99.1	97.4	83.4	99.1	82.7
WIM49482.1|KudCRBV|Ack01|USA	**77.0**	77.0	76.8	76.9	77.0		82.9	83.0	82.9	96.2	82.8	98.0
WIM49484.1|WVMV|MS2026|USA	**95.5**	95.7	86.3	95.7	95.7	77.2		97.4	99.0	83.2	97.4	82.8
WIM49485.1|WVMV|MS1211|USA	**98.3**	98.4	86.0	98.5	98.5	77.0	95.5		97.3	83.4	98.8	82.7
WIM49486.1|WVMV|MS1419|USA	**95.5**	95.6	86.2	95.6	95.6	77.3	98.9	95.5		83.3	97.2	82.8
BDS00297.1|WVMV|WVMVCeJH|KOR	**76.9**	76.8	76.6	76.9	76.9	87.3	77.3	77.0	77.2		83.4	96.3
QLK02401.1|WVMV|Ir|IRN	**98.6**	98.7	86.2	98.8	98.7	77.1	95.5	98.4	95.5	77.0		82.7
QLG97050.1|WVMV|JEBUp|KOR	**77.0**	76.8	77.1	76.8	77.1	94.1	77.3	77.1	77.3	87.3	76.8	
