# Supplementary material for: The Evolution of Wisteria Vein Mosaic Virus: A Case Study Approach to Track the Emergence of New Potyvirus Threats
Source: Pathogens. 2024 Nov 15;13(11):1001. doi: 10.3390/pathogens13111001 (PMC11597161; doi:10.3390/pathogens13111001)
Supplement: Supplementary file 1 [file pathogens-13-01001-s001.zip › Figure S2.pdf]

Figure S2. Distance matrix of pairwise MAFFT-based comparison of the complete sequence (846-849 nt; 282-283 aa) coding for the coat protein (CP) of fourteen isolates of Wisteria vein mosaic virus (WVMV) and one isolate of kudzu chlorotic ring blotch virus (KudCRBV). The percentage of nt and aa sequence identities are shown below and above the diagonal, respectively. The analysis was performed using Sequence Demarcation Tool (SDT) software, version 1.2. All sequences are identified by their accession number, virus and isolate names, country of origin. Three-letter codes are used for country names, as defined in the ISO 3166-1 standard [94]. The WVMV isolate Bari, the object of this study, is highlighted and its identity values are reported in bold. Detailed information on individual isolates can be found in Table S1.

	**OP748400.3|WVMV|Bari|ITA**	MN514947.1|WVMV|Ir|IRN	MH558668.1|WVMV|Th-W2259|IRN	OQ993365.1|WVMV|DSMZ_PV-1026|DEU	OQ731912.1|WVMV|DSMZ_PV-1105|DEU	AF484549.1|WVMV|Australia|AUS	OQ148666.1|WVMV|MS20-26|USA	OQ148668.1|WVMV|MS14-19|USA	OQ148667.1|WVMV|MS12-11|USA	PP835448.1|WVMV|Gli2|ITA	NC_007216.1|WVMV|Beijing|CHI	MK119780.1|WVMV|YZ|CHI	MT603851|WVMV|JEBU-p|KOR	LC729727|WVMV|Ce-JH|KOR	OQ148665.1|KudCRBV|Ack01|USA
**OR567543|WVMV|Bari|ITA**		**100.0**	**100.0**	**100.0**	**100.0**	**99.7**	**99.3**	**98.9**	**98.9**	**99.3**	**96.8**	**96.8**	**79.9**	**81.3**	**80.6**
MN514947.1|WVMV|Ir|IRN	**99.1**		100.0	100.0	100.0	99.7	99.3	98.9	98.9	99.3	96.8	96.8	79.9	81.3	80.6
MH558668.1|WVMV|Th-W2259|IRN	**98.9**	98.5		100.0	100.0	99.7	99.3	98.9	98.9	99.3	96.8	96.8	79.9	81.3	80.6
OQ993365.1|WVMV|DSMZ_PV-1026|DEU	**99.7**	99.2	99.1		100.0	99.7	99.3	98.9	98.9	99.3	96.8	96.8	79.9	81.3	80.6
OQ731912.1|WVMV|DSMZ_PV-1105|DEU	**99.7**	99.2	99.3	99.8		99.7	99.3	98.9	98.9	99.3	96.8	96.8	79.9	81.3	80.6
AF484549.1|WVMV|Australia|AUS	**99.4**	98.9	99.1	99.5	99.5		98.9	98.6	99.3	98.9	96.5	96.5	79.9	81.3	80.6
OQ148666.1|WVMV|MS20-26|USA	**97.4**	96.8	96.7	97.2	97.2	96.9		99.7	98.2	98.6	96.8	96.8	79.9	81.3	80.6
OQ148668.1|WVMV|MS14-19|USA	**97.4**	97.0	96.9	97.4	97.4	97.2	99.5		97.9	98.2	96.5	96.5	79.9	80.9	80.6
OQ148667.1|WVMV|MS12-11|USA	**98.8**	98.6	98.5	98.9	98.9	99.2	96.6	96.8		98.2	96.5	96.5	79.9	81.3	80.6
PP835448.1|WVMV|Gli2|ITA	**99.1**	98.4	98.2	98.9	98.9	98.7	96.8	96.8	98.1		96.1	96.1	79.2	80.6	79.9
NC_007216.1|WVMV|Beijing|CHI	**88.1**	88.3	88.3	88.1	88.1	88.3	87.7	87.7	88.4	87.9		99.3	80.2	81.6	80.6
MK119780.1|WVMV|YZ|CHI	**88.5**	89.0	88.8	88.5	88.5	88.8	87.9	87.9	89.1	88.4	98.5		80.2	81.6	80.6
MT603851|WVMV|JEBU-p|KOR	**77.4**	77.2	77.1	77.5	77.4	77.6	77.2	77.1	77.6	76.9	77.9	77.6		95.1	98.2
LC729727|WVMV|Ce-JH|KOR	**78.2**	78.1	77.9	78.1	77.9	78.2	77.8	77.7	78.4	77.8	78.2	77.9	88.2		95.1
OQ148665.1|KudCRBV|Ack01|USA	**78.2**	78.3	77.7	78.1	77.9	78.2	77.9	77.6	78.4	77.7	78.1	77.8	95.6	88.6	
