# Supplementary material for: The Evolution of Wisteria Vein Mosaic Virus: A Case Study Approach to Track the Emergence of New Potyvirus Threats
Source: Pathogens. 2024 Nov 15;13(11):1001. doi: 10.3390/pathogens13111001 (PMC11597161; doi:10.3390/pathogens13111001)
Supplement: Supplementary file 1 [file pathogens-13-01001-s001.zip › Figure S3.pdf]

Figure S3. Distance matrices of pairwise MAFFT-based comparison of the (a) complete (948-957 nt; 316-319 aa) or (b) N-terminal (1-300/309 nt; 1-100/103 aa) sequences coding for the P1 proteinase (P1-Pro) of eleven isolates of Wisteria vein mosaic virus (WVMV) and one isolate of kudzu chlorotic ring blotch virus (KudCRBV). The percentage of nt and aa sequence identities are shown below and above diagonals, respectively. The analysis was performed using Sequence Demarcation Tool (SDT) software, version 1.2. All sequences are identified by their accession number, virus and isolate names, country of origin. Three-letter codes are used for country names, as defined in the ISO 3166-1 standard [94]. The WVMV isolate Bari, the object of this study, is highlighted and its identity values are reported in bold. Detailed information on individual isolates can be found in Table S1.

a

	**OR567543|WVMV|Bari|ITA**	PP835448|WVMV|Gli2|ITA	MN514947|WVMV|Ir|IRN	OQ148667|WVMV|MS12-11	OQ148668|WVMV|MS14-19	OQ148666|WVMV|MS20-26|USA	OQ993365|WVMV|PV-1026|DEU	OQ731912|WVMV|PV-1105|DEU	NC007216|WVMV|Beijing|CHN	MT603851|WVMV|JEBU-p|KOR	LC729727|WVMV|Ce-JH|KOR	OQ148665|KudCRBV|Ack01|USA
**OR567543|WVMV|Bari|ITA**		**97.2**	**98.1**	**95.9**	**88.6**	**90.2**	**96.5**	**97.5**	**60.7**	**46.1**	**46.7**	**46.9**
PP835448|WVMV|Gli2|ITA	**97.1**		98.4	96.9	88.3	89.9	97.5	98.4	61.0	46.1	46.7	46.3
MN514947|WVMV|Ir|IRN	**97.5**	98.5		97.2	88.6	90.2	97.8	98.7	60.4	46.4	47.0	46.6
OQ148667|WVMV|MS12-11	**96.4**	97.7	97.7		88.6	89.6	96.9	97.2	60.7	46.4	47.0	46.6
OQ148668|WVMV|MS14-19	**91.1**	91.7	91.7	92.3		96.9	88.6	88.6	60.1	48.6	48.0	47.8
OQ148666|WVMV|MS20-26|USA	**91.7**	92.5	92.3	92.7	98.0		90.2	90.2	60.4	49.5	48.0	48.8
OQ993365|WVMV|PV-1026|DEU	**97.1**	98.5	98.5	97.9	92.2	92.9		97.8	61.0	47.0	47.7	46.6
OQ731912|WVMV|PV-1105|DEU	**97.3**	98.7	98.7	97.9	91.9	92.5	98.7		60.7	46.1	46.7	46.3
NC007216|WVMV|Beijing|CHN	**68.4**	68.6	68.9	68.5	68.4	68.7	68.7	68.6		45.2	45.8	45.1
MT603851|WVMV|JEBU-p|KOR	**57.8**	58.0	58.6	57.8	59.1	59.2	58.2	58.3	56.6		85.8	89.7
LC729727|WVMV|Ce-JH|KOR	**57.7**	58.5	58.4	57.7	58.5	58.8	58.3	58.0	56.9	86.5		83.4
OQ148665|KudCRBV|Ack01|USA	**57.2**	58.1	58.3	57.6	58.7	59.2	57.8	57.7	55.7	90.8	86.8	

b

	**OR567543|WVMV|Bari|ITA**	PP835448|WVMV|Gli2|ITA	MN514947|WVMV|Ir|IRN	OQ148667|WVMV|MS12-11	OQ148668|WVMV|MS14-19	OQ148666|WVMV|MS20-26|USA	OQ993365|WVMV|PV-1026|DEU	OQ731912|WVMV|PV-1105|DEU	NC007216|WVMV|Beijing|CHN	MT603851|WVMV|JEBU-p|KOR	LC729727|WVMV|Ce-JH|KOR	OQ148665|KudCRBV|Ack01|USA
**OR567543|WVMV|Bari|ITA**		**95.0**	**96.0**	**94.0**	**88.0**	**89.0**	**94.0**	**94.0**	**56.0**	**29.1**	**32.0**	**30.2**
PP835448|WVMV|Gli2|ITA	**96.0**		97.0	97.0	89.0	90.0	97.0	97.0	56.0	30.1	33.0	30.2
MN514947|WVMV|Ir|IRN	**97.3**	98.0		96.0	88.0	89.0	96.0	96.0	55.0	30.1	33.0	30.2
OQ148667|WVMV|MS12-11	**95.7**	96.3	97.0		88.0	89.0	96.0	96.0	55.0	30.1	33.0	30.2
OQ148668|WVMV|MS14-19	**92.0**	92.0	92.7	93.0		97.0	88.0	88.0	53.0	30.1	30.1	29.3
OQ148666|WVMV|MS20-26|USA	**92.3**	92.3	93.0	93.3	99.0		89.0	89.0	53.0	30.1	30.1	29.3
OQ993365|WVMV|PV-1026|DEU	**96.0**	97.3	98.0	96.3	92.7	93.0		96.0	55.0	30.1	33.0	30.2
OQ731912|WVMV|PV-1105|DEU	**96.7**	98.0	98.7	97.0	92.7	93.0	98.0		56.0	29.1	32.0	29.3
NC007216|WVMV|Beijing|CHN	**68.0**	67.7	67.7	68.0	67.3	67.3	67.7	68.0		25.2	28.2	26.4
MT603851|WVMV|JEBU-p|KOR	**40.7**	40.3	42.0	41.3	40.3	40.3	41.3	41.7	39.3		80.0	83.5
LC729727|WVMV|Ce-JH|KOR	**39.3**	39.7	40.0	40.0	40.0	40.0	40.0	39.7	40.7	84.0		74.8
OQ148665|KudCRBV|Ack01|USA	**39.2**	40.1	40.5	40.5	40.1	40.1	40.5	40.1	39.2	88.0	82.5	
