# Supplementary material for: The Evolution of Wisteria Vein Mosaic Virus: A Case Study Approach to Track the Emergence of New Potyvirus Threats
Source: Pathogens. 2024 Nov 15;13(11):1001. doi: 10.3390/pathogens13111001 (PMC11597161; doi:10.3390/pathogens13111001)
Supplement: Supplementary file 1 [file pathogens-13-01001-s001.zip › Figure S4.pdf]

Figure S4. Distance matrix of pairwise MAFFT-based comparison of the sequence of a 525 nt (175 aa) long fragment spanning the genes for the nuclear inclusion b (NIb) and capsid proteins of ten isolates of Wisteria vein mosaic virus (WVMV) found in Italy. The percentage of nt and aa sequence identities are shown below and above the diagonal, respectively. The analysis was performed using Sequence Demarcation Tool (SDT) software, version 1.2. All sequences are identified by their accession number, species and isolate names, country of origin. Three-letter codes are used for country names, as defined in the ISO 3166-1 standard [94]. The WVMV isolate Bari, the object of this study, is highlighted and its identity values are reported in bold. Detailed information on individual isolates can be found in Table S1.

	**OR567543|WVMV|Bari|ITA**	OM417219.1|WVMV|Sar-5|ITA	OM417220.1|WVMV|Sar-6|ITA	OM417221.1|WVMV|Sar-7|ITA	OM417222.1|WVMV|Sar-8|ITA	OM417223.1|WVMV|Sar-9|ITA	OM417224.1|WVMV|Sar-10|ITA	OM417225.1|WVMV|Sar-11|ITA	OM417226.1|WVMV|Sar-12|ITA	PP835448|WVMV|Gli2|ITA
**OR567543|WVMV|Bari|ITA**		**94.3**	100.0	98.9	100.0	100.0	100.0	99.4	100.0	98.3
OM417219.1|WVMV|Sar-5|ITA	**87.4**		94.3	94.3	94.3	94.3	94.3	94.8	94.3	92.5
OM417220.1|WVMV|Sar-6|ITA	**99.4**	87.8		98.9	100.0	100.0	100.0	99.4	100.0	98.3
OM417221.1|WVMV|Sar-7|ITA	**96.6**	86.7	96.6		98.9	98.9	98.9	98.3	98.9	97.1
OM417222.1|WVMV|Sar-8|ITA	**99.8**	87.4	99.6	96.6		100.0	100.0	99.4	100.0	98.3
OM417223.1|WVMV|Sar-9|ITA	**99.4**	87.4	99.2	96.2	99.6		100.0	99.4	100.0	98.3
OM417224.1|WVMV|Sar-10|ITA	**99.8**	87.4	99.6	96.6	100.0	99.6		99.4	100.0	98.3
OM417225.1|WVMV|Sar-11|ITA	**98.7**	88.6	98.5	96.2	98.9	98.5	98.9		99.4	97.7
OM417226.1|WVMV|Sar-12|ITA	**99.6**	87.6	99.4	96.8	99.8	99.4	99.8	99.1		98.3
PP835448|WVMV|Gli2|ITA	**98.5**	86.3	98.3	95.6	98.7	98.3	98.7	97.5	98.5	
