# Supplementary material for: The Evolution of Wisteria Vein Mosaic Virus: A Case Study Approach to Track the Emergence of New Potyvirus Threats
Source: Pathogens. 2024 Nov 15;13(11):1001. doi: 10.3390/pathogens13111001 (PMC11597161; doi:10.3390/pathogens13111001)

Figure S5. Plot of the SLAC (Single-Likelihood Ancestor Counting) analysis conducted on the coding sequence of the coat protein of Wisteria vein mosaic virus (WVMV) and kudzu chlorotic ring blotch virus (KudCRBV) isolates. The SLAC algorithm was accessed in the Datamonkey [67] web-based suite. MAFFT-aligned nucleotide sequences (846-849 nt) were used as input for the analysis. The site positions reported in the x-axis refer to codon triplets. The y-axis represents the P[d*N*/d*S*<1] binomial probability that the inferred synonymous substitution (*S*) is no less than the observed value, with P_S probability of success. Points below the line (*p*-value threshold=0.1) represent sites under negative selection. Fourteen isolates of WVMV and one isolate of KudCRBV were included in the analysis. Detailed information on individual isolates is available in Table S1.

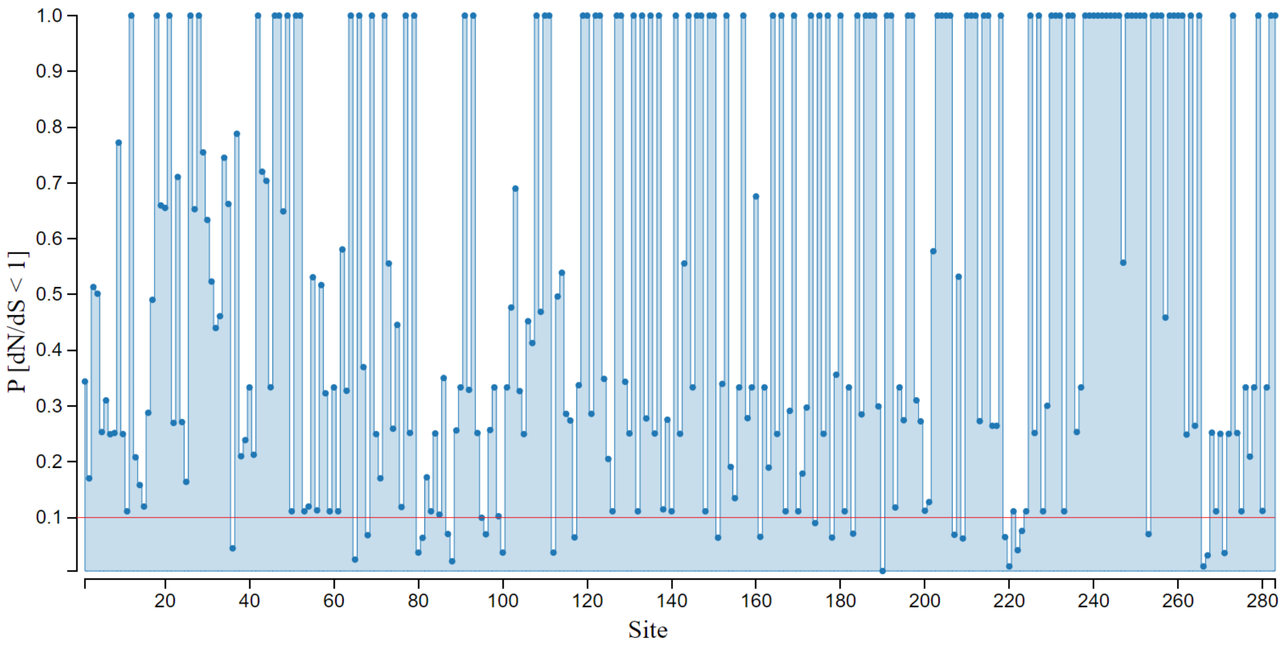

Supplement: Supplementary file 1 [file pathogens-13-01001-s001.zip › Figure S5.pdf]
