# Supplementary material for: The Evolution of Wisteria Vein Mosaic Virus: A Case Study Approach to Track the Emergence of New Potyvirus Threats
Source: Pathogens. 2024 Nov 15;13(11):1001. doi: 10.3390/pathogens13111001 (PMC11597161; doi:10.3390/pathogens13111001)
Supplement: Supplementary file 1 [file pathogens-13-01001-s001.zip › Table S2.pdf]

Table S2. Summary of BLASTX analyses of contigs *de novo* assembled from reads generated by high-throughput sequencing (HTS) of the *Wisteria sinensis* tree affected by Wisteria mosaic disease (WMD).

Virus genes	Contig number	Average length (nt)	Min length (nt)	Max length (nt)	Average identity (%)
Potyvirus	133	250.17	101	9,694	91.4
Badnavirus	2	371.5	333	410	82.7
Deltapartitivirus	1	397.0	397	397	97.6
Alphaendornavirus	2	165.0	130	200	51.1

The *de novo* assembly was built with MEGAHIT v. 1.2.9 assembler. The average identity values refer to the local BLASTX alignment on RefSeq viral proteins, performed with the NCBI BLAST+ search tool. The expected value (E-value) cutoff was set at 0.001. Contigs showing alignment hits shorter than 100 nt in BLASTN search against RefSeq nucleotides were preliminarily excluded from this analysis.
