# Supplementary material for: The Evolution of Wisteria Vein Mosaic Virus: A Case Study Approach to Track the Emergence of New Potyvirus Threats
Source: Pathogens. 2024 Nov 15;13(11):1001. doi: 10.3390/pathogens13111001 (PMC11597161; doi:10.3390/pathogens13111001)
Supplement: Supplementary file 1 [file pathogens-13-01001-s001.zip › Table S3.pdf]

Table S3. Genomic structure of Wisteria vein mosaic virus (WVMV), isolate Bari with putatively encoded proteins and predicted cleavage sites.

**Genomic region**	**Putative product**	**Position (nt)**		**Size**		**Cleavage site**
		**start**	**end**	**nt**	**aa**	
5′-UTR		1	163	163	-	-
ORF1	polyprotein	164	9,442	9,279	3,092	-
	P1-Pro	164	1,114	951	317	-
	HC-Pro	1,115	2,485	1,371	457	IQH**Y/S**
	P3	2,486	3,526	1,041	347	YRV**G/G**
	6K1	3,527	3,682	156	52	VSI**Q/A**
	CI	3,683	5,584	1902	634	VKI**Q/S**
	6K2	5,585	5,743	159	53	VQL**Q/S**
	VPg	5,744	6,313	570	190	VST**Q/G**
	NIa-Pro	6,314	7,042	729	243	VEV**E/S**
	NIb	7,043	8,593	1551	517	VSV**Q/S**
	CP	8,594	9,442	849	282	VSL**Q/S**
ORF2	*pipo* protein	2,941	3,168	228	75	-
3′-UTR		9,443	9,694	252	-	

P1-Pro: P1 proteinase; **HC-Pro:** helper component proteinase; **P3:** P3 protein; **6K1:** 6K1 protein; **CI:** cylindrical inclusion; **6K2:** 6K2 protein; **VPg:** viral protein genome-linked; **NIa-Pro:** nuclear inclusion proteinase a; **NIb:** nuclear inclusion body b protein; **CP:** coat protein; **pipo:** Pretty Interesting Potyviridae ORF protein. Cleavage sites were predicted according to [33] and using the ExPASy PeptideCutter tool [34].
