# Supplementary material for: The Evolution of Wisteria Vein Mosaic Virus: A Case Study Approach to Track the Emergence of New Potyvirus Threats
Source: Pathogens. 2024 Nov 15;13(11):1001. doi: 10.3390/pathogens13111001 (PMC11597161; doi:10.3390/pathogens13111001)
Supplement: Supplementary file 1 [file pathogens-13-01001-s001.zip › Table S4.pdf]

Table S4. List of conserved domains common to known potyviruses, identified in the polyprotein of Wisteria vein mosaic virus (WVMV) isolate Bari.

Name	Accession	pfam entry	Description	Position (aa)	E-value
ps-ssRNAv_Potyviridae_RdRp	cd23175	n.a.	catalytic core domain of RdRp	2455-2690	8.52e-173
Peptidase_C6 superfamily	cl20022	00851	helper component proteinase	342-774	1.13e-108
Poty_coat superfamily	cl02961	00767	Potyvirus coat protein	2858-3087	1.66e-94
Poty_PP superfamily	cl07169	08440	Potyviridae polyprotein	1558-1830	4.21e-64
Peptidase_C4 superfamily	cl24133	00863	peptidase family C4	2050-2282	1.25e-54

n.a.: not available, due to the limited number of isolates available. Conserved domains were identified using the NCBI Conserved Domain search tool.
