# Supplementary material for: The Evolution of Wisteria Vein Mosaic Virus: A Case Study Approach to Track the Emergence of New Potyvirus Threats
Source: Pathogens. 2024 Nov 15;13(11):1001. doi: 10.3390/pathogens13111001 (PMC11597161; doi:10.3390/pathogens13111001)
Supplement: Supplementary file 1 [file pathogens-13-01001-s001.zip › Table S6.pdf]

Table S6. Putative recombination events identified by the detection algorithms of the RDP4 software, v. 4.101, within the coding sequence (CDS) region of Wisteria vein mosaic (WVMV) and kudzu chlorotic ring blotch virus (KudCRBV) isolates.

**Breakpoint position (nt, relative to CDS region)**		**Recombinant sequence (accession)**	**Parental sequences (accession)**	
Beginning	End		Major	Minor
2032	2302	WVMV JEBU-p (MT603851)	KudCRBV Ack01 (OQ148665)	WVMV Ce-JH (LC729727)

**Detection algorithm (*p*-value)**								
**RDP**	**GENECONV**	**BootScan**	**MaxChi**	**Chimaera**	**SiScan**	**PhylPro**	**LARD**	**3Seq**
8.71×10^{-6}	9.18×10^{-3}	6.92×10^{-5}	$1.55 \cdot \times 10^{-2}$	4.46×10^{-4}	ns	ns	ns	2.99×10^{-5}

ns: not significant (*p*-value > 0.05).
