# Supplementary material for: The Evolution of Wisteria Vein Mosaic Virus: A Case Study Approach to Track the Emergence of New Potyvirus Threats
Source: Pathogens. 2024 Nov 15;13(11):1001. doi: 10.3390/pathogens13111001 (PMC11597161; doi:10.3390/pathogens13111001)
Supplement: Supplementary file 1 [file pathogens-13-01001-s001.zip › Table S7.pdf]

Table S7. Numerical summary of the dating analysis conducted on the coding sequence of Wisteria vein mosaic virus (WVMV) and kudzu chlorotic ring blotch virus (KudCRBV) isolates.

Node Label	Node ID	Descendant Node 1	Descendant Node 2	Divergence Time (CE)	CI Lower	CI Upper	Sampling Time
NC006262 WMV Fr FRA	1	-	-	-	-	-	2004
OR567543 WVMV Bari ITA	2	-	-	-	-	-	2022
PP835448 WVMV Gli2 ITA	3	-					1996
NC007216 WVMV Beijing CHN	4	-	-	-	-	-	2004
OQ731912 WVMV PV-1105 DEU	5	-	-	-	-	-	2013
OQ993365 WVMV PV-1026 DEU	6	-	-	-	-	-	2011
OQ148665 KudCRBV Ack01 USA	7	-	-	-	-	-	2009
OQ148666 WVMV MS20-26 USA	8	-	-	-	-	-	2021
OQ148667 WVMV MS12-11 USA	9	-	-	-	-	-	2021
OQ148668 WVMV MS14-19 USA	10	-	-	-	-	-	2020
LC729727 WVMV Ce-JH KOR	11	-	-	-	-	-	2022
MN514947 WVMV Ir IRN	12	-	-	-	-	-	2019
MT603851 WVMV JEBU-p KOR	13	-	-	-	-	-	2016
	14	7	13	**1981**	1952	1996	-
-	15	8	10	**2011**	1986	2017	-
-	16	5	12	**1999**	1964	2012	-
-	17	16	3	**1996**	1964	1996	-
-	18	17	2	**1996**	1964	1996	-
-	19	18	6	**1996**	1964	1996	-
-	20	19	9	**1996**	1964	1995	-
-	21	20	15	**1981**	1942	1991	-
-	22	21	4	**1881**	1791	1930	-
-	23	22	24	**1664**	1454	1792	-
-	24	11	14	**1930**	1877	1961	-
outgroup	25	23	1	-	-	-	-

The node IDs are those shown in the time tree outlined in Figure 5 and inferred by applying the RelTime with Dated Tips (RTDT) method [53]. The time tree was computed in Mega11, using sampling tip dates for 11 isolates of WVMV and one isolate of KudCRBV. An isolate of watermelon mosaic virus (WMV) was used as an outgroup. All sequences are identified by their accession number, virus and isolate names, and country of origin. Three-letter codes are used for country names, as defined in the ISO 3166-1 standard [94]. Detailed information on individual isolates is available in Table S1. All dates in the table are rounded to the relevant year. **CE:** Common Era. **CI:** confidence interval.
