# Supplementary material for: The Evolution of Wisteria Vein Mosaic Virus: A Case Study Approach to Track the Emergence of New Potyvirus Threats
Source: Pathogens. 2024 Nov 15;13(11):1001. doi: 10.3390/pathogens13111001 (PMC11597161; doi:10.3390/pathogens13111001)
Supplement: Supplementary file 1 [file pathogens-13-01001-s001.zip › Table S8.pdf]

Table S8. Codons found under negative selection according to a SLAC (Single-Likelihood Ancestor Counting) analysis conducted on the coding sequence of the coat protein of Wisteria vein mosaic virus (WVMV) and kudzu chlorotic ring blotch virus (KudCRBV) isolates.

Site	E*S*	E*N*	*S*	*N*	P[*S*]	d*S*	d*N*	P [d*N*/d*S* < 1]
36	0.79	2.21	4.00	2.00	0.26	5.07	0.90	0.04
65	0.87	2.13	3.00	0.00	0.29	3.44	0.00	0.02
68	0.74	2.10	2.00	0.00	0.26	2.69	0.00	0.07
80	1.00	2.00	3.00	0.00	0.33	3.00	0.00	0.04
81	0.76	2.24	2.00	0.00	0.25	2.64	0.00	0.06
87	0.82	2.09	3.00	1.00	0.28	3.67	0.48	0.07
88	0.77	2.00	3.00	0.00	0.28	3.89	0.00	0.02
95	1.66	1.29	4.00	0.00	0.56	2.42	0.00	0.10
96	0.79	2.21	2.00	0.00	0.26	2.52	0.00	0.07
100	1.00	2.00	3.00	0.00	0.33	3.00	0.00	0.04
112	1.00	2.00	3.00	0.00	0.33	3.00	0.00	0.04
117	0.74	2.16	2.00	0.00	0.25	2.72	0.00	0.06
151	0.76	2.24	2.00	0.00	0.25	2.64	0.00	0.06
161	0.73	2.12	2.00	0.00	0.26	2.75	0.00	0.07
174	0.90	2.10	2.00	0.00	0.30	2.22	0.00	0.09
178	0.76	2.24	2.00	0.00	0.25	2.63	0.00	0.06
183	0.77	2.13	2.00	0.00	0.27	2.59	0.00	0.07
190	0.76	2.24	4.00	0.00	0.25	5.29	0.00	0.00
207	0.79	2.21	2.00	0.00	0.26	2.54	0.00	0.07
209	0.75	2.25	2.00	0.00	0.25	2.67	0.00	0.06
219	0.74	2.16	2.00	0.00	0.25	2.71	0.00	0.06
220	1.00	2.00	4.00	0.00	0.33	4.00	0.00	0.04
222	1.00	1.89	3.00	0.00	0.35	3.00	0.00	0.04
223	0.78	2.06	2.00	0.00	0.28	2.55	0.00	0.08
253	0.79	2.21	2.00	0.00	0.26	2.52	0.00	0.07
266	1.00	2.00	4.00	0.00	0.33	4.00	0.00	0.01
267	0.95	2.03	3.00	0.00	0.32	3.17	0.00	0.03
271	0.73	1.47	3.00	0.00	0.33	4.14	0.00	0.04

The SLAC algorithm was accessed in the Datamonkey [67] web-based suite. The *p*-value threshold was set at 0.1. MAFFT-aligned nucleotide sequences (846-849 nt) were used as input for the analysis. The site positions refer to codon triplets. Fourteen isolates of WVMV and one isolate of KudCRBV were included in the analysis. Detailed information on individual isolates is available in Table S1. **E*S*:** expected synonymous sites; **E*N*:** expected non-synonymous sites; ***S*:** inferred synonymous substitutions; ***N*:** inferred non-synonymous substitutions; **P[*S*]:** expected proportion of synonymous sites; **d*S*:** inferred synonymous substitution rate; **d*N*:** inferred non-synonymous substitution rate; **P[d*N*/d*S*<1]:** binomial probability that *S* is no less than the observed value, with P_S probability of success.
